# ARID1A and PI3-kinase pathway mutations in the endometrium drive epithelial transdifferentiation and collective invasion

**DOI:** 10.1038/s41467-019-11403-6

**Published:** 2019-08-07

**Authors:** Mike R. Wilson, Jake J. Reske, Jeanne Holladay, Genna E. Wilber, Mary Rhodes, Julie Koeman, Marie Adams, Ben Johnson, Ren-Wei Su, Niraj R. Joshi, Amanda L. Patterson, Hui Shen, Richard E. Leach, Jose M. Teixeira, Asgerally T. Fazleabas, Ronald L. Chandler

**Affiliations:** 1 Department of Obstetrics, Gynecology, and Reproductive Biology, College of Human Medicine, Michigan State University, Grand Rapids, MI 49503 USA; 2 Genomics Core Facility, Van Andel Research Institute, Grand Rapids, MI 49503 USA; 3 Bioinformatics and Biostatistics Core Facility, Van Andel Research Institute, Grand Rapids, MI 49503 USA; 4 Center for Epigenetics, Van Andel Research Institute, Grand Rapids, MI 49503 USA; 5 Department of Women’s Health, Spectrum Health System, Grand Rapids, MI 49341 USA

**Keywords:** Cancer models, Endometrial cancer, Endometrial cancer, Cancer epigenetics

## Abstract

ARID1A and PI3-Kinase (PI3K) pathway alterations are common in neoplasms originating from the uterine endometrium. Here we show that monoallelic loss of ARID1A in the mouse endometrial epithelium is sufficient for vaginal bleeding when combined with PI3K activation. Sorted mutant epithelial cells display gene expression and promoter chromatin signatures associated with epithelial-to-mesenchymal transition (EMT). We further show that ARID1A is bound to promoters with open chromatin, but ARID1A loss leads to increased promoter chromatin accessibility and the expression of EMT genes. PI3K activation partially rescues the mesenchymal phenotypes driven by ARID1A loss through antagonism of ARID1A target gene expression, resulting in partial EMT and invasion. We propose that ARID1A normally maintains endometrial epithelial cell identity by repressing mesenchymal cell fates, and that coexistent ARID1A and PI3K mutations promote epithelial transdifferentiation and collective invasion. Broadly, our findings support a role for collective epithelial invasion in the spread of abnormal endometrial tissue.

## Abstract (web summary)

PIK3CA mutations and ARID1A loss co-exist in endometrial neoplasms. Here, the authors show that these co-mutations drive gene expression profiles correlated with differential chromatin accessibility and ARID1A binding in the endometrial epithelium, resulting in partial EMT and myometrial invasion.

## Introduction

The endometrium is the dynamic inner layer of the uterus, composed of stroma and epithelial cells that undergo monthly proliferation, differentiation, and shedding throughout the menstrual cycle in reproductive age women^1^. Disruption of normal endometrial processes results in a number of pathologies, including endometrial hyperplasia, endometrial cancer (EC)^2^, endometriosis^3^, adenomyosis^4^, and endometriosis-associated ovarian cancer (EAOC)^5^. An estimated 63,230 women will be diagnosed with EC this year^6^, making it the most commonly diagnosed gynecologic malignancy. Furthermore, EC incidence is rising due to the increasing prevalence of obesity^2,7^.

The SWI/SNF chromatin remodeling complex is mutated in >20% of all human cancers^8,9^, and the ARID1A (BAF250A) subunit is particularly prone to mutation in gynecologic cancer^5,10–13^. ARID1A mutations are found in 40% of low-grade EC^12^, while ARID1A protein expression is lost in 26–29% of low-grade and 39% of high-grade EC^13^. ARID1A loss is observed in focal areas of atypical endometrial hyperplasia^14^, indicating clonal loss. Loss of ARID1A in complex atypical hyperplasia is associated with malignant transformation and concurrent EC^15^. ARID1A mutations are observed in 11% of endometriosis and >30% of EAOCs^3,5,16,17^. These data support a tumor suppressor role for ARID1A-containing SWI/SNF complexes in neoplasms originating from the endometrium.

Among highly mutated tumor suppressor genes, ARID1A is unique because ARID1A knockout mice are embryonic lethal in the heterozygous state^18^, while other tumor suppressor genes (e.g., TP53) are non-essential for mouse development^19^. ARID1A-null embryos die at embryonic day (E) E6.5^18^, while DNA-binding defective ARID1A^V1068G^ mutant embryos die around E10^20^. ARID1A mutations are often nonsense and result in a frameshift of the open-reading frame^10^, a characteristic of many tumor suppressors.

Mutations leading to PI3K/AKT pathway upregulation are frequent in EC^21^, with 84% of patients displaying mutations in PIK3CA, PIK3R1, or PTEN^22^. PIK3CA mutations commonly co-occur with ARID1A loss in EC^23^. However, PIK3CA mutations have been observed in normal endometrium^17^. Missense mutations of PIK3CA are common in complex atypical hyperplasia, and PIK3CA mutation has been identified as an early event in endometrial carcinogenesis^24^.

Genetically engineered mouse models (GEMMs) offer the opportunity to study gynecologic pathologies in vivo^25–28^. ARID1A loss in the mouse ovarian surface epithelium drives tumorigenesis when paired with PTEN loss or PIK3CA^H1047R^ mutation^29,30^. In this study, we utilize lactotransferrin-Cre (*LtfCre*) to target ARID1A mutations and PIK3CA^H1047R^ directly to the endometrial epithelium. Utilizing the *Arid1a*^*fl*^ and *Arid1a*^*V1068G*^ alleles, we develop an allelic series of loss of function ARID1A mutations in the endometrium, each with increasing severity. We employ genome-wide approaches to profile gene expression and chromatin accessibility of sorted endometrial epithelial cells in vivo and identified chromatin accessibility changes at promoters upon ARID1A loss, which correlate with changes in transcription. Using chromatin immunoprecipitation sequencing (ChIP-seq), we show that ARID1A binding correlates with chromatin accessibility and is associated with gene expression changes upon loss of ARID1A. We utilize human endometrial epithelial cells to elucidate the consequences of ARID1A loss and PIK3CA^H1047R^ in vitro, and discover a mechanism by which ARID1A and PIK3CA mutations result in a partial EMT phenotype capable of collective invasion into the uterine myometrium. In this context, we characterize the role of ARID1A in epithelial cell identity of the endometrium.

## Results

### ARID1A is haploinsufficient in the endometrial epithelium

**Fig. 1:**
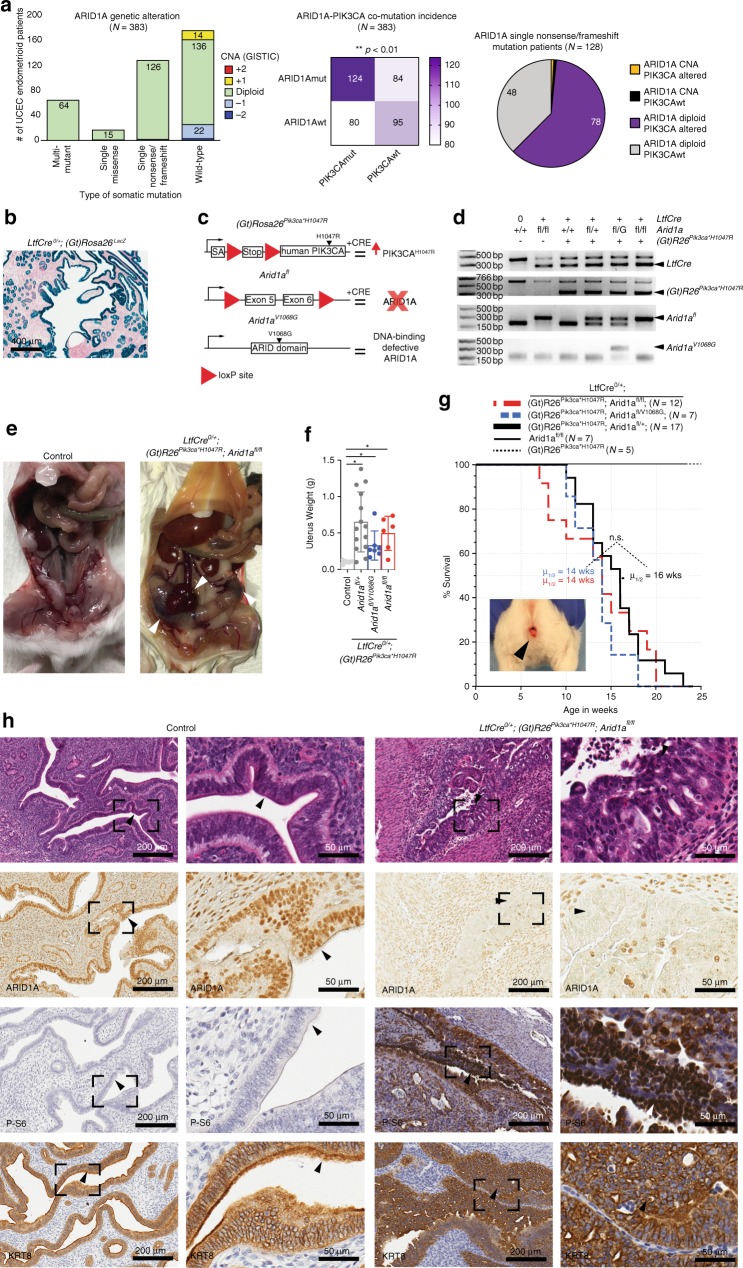
Development of genetic mouse models representing an allelic series of ARID1A mutations in the endometral epithelium. **a** UCEC endometrioid patient ARID1A alteration status and co-incidence with PIK3CA mutation, taken from TCGA-UCEC dataset. **b** LacZ expression (blue) is specific to the endometrial epithelium. Sections were counter-stained with nuclear fast red (scale bar = 400 μm). **c** Diagram of mutant alleles utilized in this study. **d** PCR genotyping results to detect *LtfCre*^*0/+*^, (Gt)R26Pik3ca^*H1047R^*, Arid1a*^*fl*^, and *Arid1a*^*V1068G*^. **e** Representative gross images of mice at time of sacrifice due to vaginal bleeding. White arrows indicate tumors. Size of uterine tumor varies within genotype at time of sacrifice. **f** Weight of semi-dry mouse uterus by genotype. Control (*N* = 5), *LtfCre*^*0/+*^; (Gt)R26Pik3ca^*H1047R^; *Arid1a*^*fl/+*^ (*N* = 14), *LtfCre*^*0/+*^; (Gt)R26Pik3ca^*H1047R^*; Arid1a*^*fl/V1068G*^ (*N* = 7), *LtfCre*^*0/+*^; (Gt)R26Pik3ca^*H1047R^*; Arid1a*^*fl/fl*^ (*N* = 6) (mean ± s.d; * *p* < 0.05, unpaired *t*-test, two-tailed). **g** Survival of mice, based on time until vaginal bleeding. (Gt)R26Pik3ca^*H1047R^ (*N* = 5), *Arid1a*^*fl/fl*^ (*N* = 7), (Gt)R26Pik3ca^*H1047R^; *Arid1a*^*fl/+*^ (*N* = 17), (Gt)R26Pik3ca^*H1047R^*; Arid1a*^*fl/V1068G*^ (*N* = 7), (Gt)R26Pik3ca^*H1047R^*; Arid1a*^*fl/fl*^ (*N* = 12). Mice succumb to vaginal bleeding (sample image inset) at a median (*μ*_1/2_) of 16 weeks (*LtfCre*^*0/+*^; (Gt)R26Pik3ca^*H1047R^ *Arid1a*^*fl/fl*^) or 14 weeks (*LtfCre*^*0/+*^; (Gt)R26Pik3ca^*H1047R^*; Arid1a*^*fl/+*^, and *LtfCre*^*0/+*^; (Gt)R26Pik3ca^*H1047R^*; Arid1a*^*fl/V1068G*^), without a significant difference between these genotypes. *LtfCre*^*0/+*^ mice harboring *Arid1a*^*fl/fl*^ or (Gt)R26Pik3ca^*H1047R^ alone did not develop vaginal bleeding. **h** H&E staining and IHC for ARID1A, P-S6 and KRT8 (*N* ≥ 2) of the endometrium at 5 × (scale bar = 200 μm) and 20 × (scale bar = 50 μm) magnification, with x20 magnifications representing portion panel to the right surrounded by black box. ARID1A expression is lost in the endometrial epithelium of *LtfCre*^*0/+*^; (Gt)R26Pik3ca^*H1047R^; *Arid1a*^*fl/fl*^ mice. P-S6 is shown as marker of AKT pathway activation; KRT8 as a marker of endometrial epithelium arrows indicate endometrial epithelium

ARID1A has been hypothesized to function as a haploinsufficient tumor suppressor^31^. To explore this further, we utilized publicly available Uterine Corpus Endometrial Carcinoma (UCEC) mutation and copy-number datasets from The Cancer Genome Atlas (TCGA). Most endometrioid EC patients with ARID1A mutations (either single or multiple hits) show no detectable copy-number alterations at the ARID1A locus, with 33% of all patients having a single nonsense mutation and normal ploidy at ARID1A (Fig. 1a). Co-existing PIK3CA mutation was significantly associated with ARID1A mutation, and a majority (61%) of heterozygous ARID1A tumors also have PIK3CA alterations (Fig. 1a). These data demonstrate that 20% of endometrioid EC patients are genetically heterozygous for ARID1A mutations and carry PIK3CA alterations.

To induce CRE in the mouse endometrial epithelium, we utilized *LtfCre* (*Tg(Ltf-iCre)14Mmul). LtfCre* induction occurs naturally as females undergo sexual maturity, becoming fully active by 60 days^32^ (Fig. 1b). To investigate the consequence of ARID1A loss in the endometrial epithelium, we bred *LtfCre*^*0/+*^ mice to mice with an *Arid1a*^*fl*^ allele, permitting conditional knockout of ARID1A upon CRE expression (Fig. 1c)^30^. Genotyping by PCR confirmed expression of each allele (Fig. 1d). We observed no gross phenotypes in *LtfCre*^*0/+*^; *Arid1a*^*fl/fl*^ mice (Supplementary Fig. 1a). Previously, we found (Gt)R26Pik3ca^*H1047R^ to be a potent driver of epithelial ovarian tumors when combined with *Arid1a*^*fl/fl* 30^. (Gt)R26Pik3ca^*H1047R^ provides conditional expression of the oncogenic PIK3CA^H1047R^ mutation (Fig. 1c)^33^. No gross phenotypes were observed in *LtfCre*^*0/+*^; (Gt)R26Pik3ca^*H1047R^ (Supplementary Fig. 1a), as previously described in the endometrial epithelium^34^. Therefore, we bred *LtfCre* mice with mice harboring (Gt)R26Pik3ca^*H1047R^, *Arid1a*^*fl*^, and *Arid1a*^*V1068G*^ (DNA-binding domain defective ARID1A mutant, Fig. 1c)^20^ to develop an allelic series with increasing ARID1A mutational burden in the endometrial epithelium.

Abnormal vaginal bleeding is a prominent symptom of endometrial dysfunction in humans. *LtfCre*^*0/+*^; (Gt)R26Pik3ca^*H1047R^; *Arid1a*^*fl/fl*^ mice were sacrificed after a median age of 14 weeks due to vaginal bleeding and uterine tumors (Fig. 1e, g). Surprisingly, homozygous ARID1A loss was not required for vaginal bleeding, as *LtfCre*^*0/+*^; (Gt)R26Pik3ca^*H1047R^*; Arid1a*^*fl/+*^ mice developed endometrial lesions and vaginal bleeding (Fig. 1e, g). For both *LtfCre*^*0/+*^; (Gt)R26Pik3ca^*H1047R^*; Arid1a*^*fl/+*^, and *LtfCre*^*0/+*^; (Gt)R26Pik3ca^*H1047R^*; Arid1a*^*fl/V1068G*^ mice, median uterus weight, and survival were not significantly different from *LtfCre*^*0/+*^; (Gt)R26Pik3ca^*H1047R^*; Arid1a*^*fl/fl*^ (Fig. 1f, g). ARID1A loss and PI3K pathway activation (via phospho-S6 ribosomal protein, P-S6, expression) were determined by immunohistochemistry, while Cytokeratin 8 (KRT8) labeled the endometrial epithelium (Fig. 1h and Supplementary Fig. 1b). *LtfCre*^*0/+*^; (Gt)R26Pik3ca^*H1047R^*; Arid1a*^*fl/+*^, *LtfCre*^*0/+*^; (Gt)R26Pik3ca^*H1047R^*; Arid1a*^*fl/V1068G*^, and *LtfCre*^*0/+*^; (Gt)R26Pik3ca^*H1047R^*; Arid1a*^*fl/fl*^ showed evidence of widespread atypical endometrial hyperplasia and nuclear atypia, including glandular crowding and abnormal cytologic features (Fig. 1h and Supplementary Fig. 1b). Endometrial tumors were moderately to poorly differentiated, with areas of solid and cribiform architecture (Fig. 1h). In one mouse, we observed visible lung metastasis (Supplementary Fig. 1c), a site of metastasis in some EC patients. In the *LtfCre*^*0/+*^; (Gt)R26Pik3ca^*H1047R^*; Arid1a*^*fl/fl*^ endometrial epithelium we observed downregulation of estrogen receptor-α (ESR1) and loss of the progesterone receptor, suggesting changes to steroid hormone regulation (Supplementary Fig. 2a). Impaired steroid hormone regulation indicates poor prognosis in EC^35^.

### Mutant endometrial epithelium show hallmarks of EMT

**Fig. 2:**
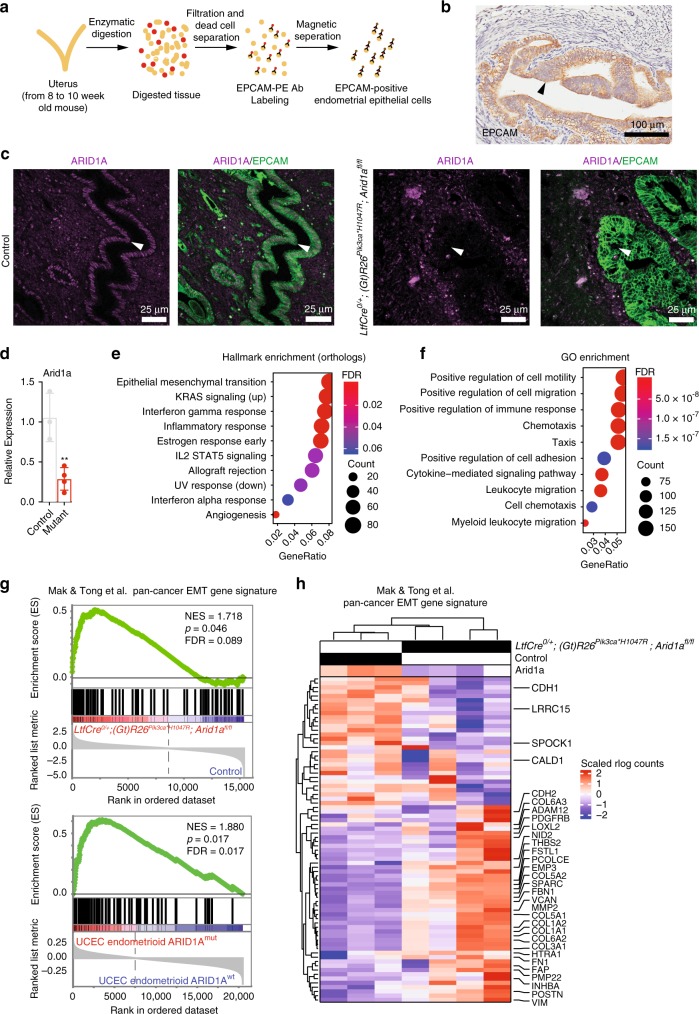
RNA-seq analysis of EPCAM-positive endometrial epithelial cells isolated via magnetic sorting. **a** Schematic of EPCAM isolation using anti-EPCAM-PE antibody and anti-PE microbeads. **b** EPCAM is expressed in the endometrial epithelium of a *LtfCre*^*0/+*^; (Gt)R26Pik3ca^*H1047R^*; Arid1a*^*fl/fl*^ mouse by IHC (*N* = 3). Arrows indicate endometrial epithelium (scale bar = 100 μm). **c** IF staining of EPCAM and ARID1A in mouse endometrium (*N* ≥ 3). Arrows indicate endometrial epithelium (scale bar = 25 μm). **d** qPCR analysis of *Arid1a* gene expression of isolated control (*N* = 3, pooled groups of six mice) and *LtfCre*^*0/+*^; (Gt)R26Pik3ca^*H1047R^*; Arid1a*^*fl/fl*^ (mutant) (*N* = 4, single mice) cells (mean ± s.d; **p < 0.01, unpaired *t*-test, two-tailed). **e**, **f** Pathway enrichment analysis on human orthologs of differentially expressed genes between *LtfCre*^*0/+*^; (Gt)R26Pik3ca^*H1047R^*; Arid1a*^*fl/fl*^, and control mice (FDR < 0.05; 3481 genes) for mSigDb Hallmark pathways (**e**) and Gene Ontology (GO) Biological Process terms (**f**). **g** GSEA plots showing significance of Mak et al. pan-cancer EMT signature upregulation within *LtfCre*^*0/+*^; (Gt)R26Pik3ca^*H1047R^*; Arid1a*^*fl/fl*^ compared to control and UCEC ARID1A^mut^ patients compared to ARID1A^wt^. **h** Hierarchical clustering of 77 genes within the Mak et al. pan-cancer EMT signature between control and mutant purified endometrium. Genes found in the Hallmark EMT pathway, and CDH1, are identified

To profile in vivo gene expression changes in mutant endometrial epithelium at an early stage of transformation, we devised an enzymatic digestion and magnetic isolation protocol to positively enrich epithelial populations (Fig. 2a). Endometrial epithelial cells express EPCAM (Fig. 2b), and EPCAM expression is not altered in the hyperplastic endometrium of *LtfCre*^*0/+*^; (Gt)R26Pik3ca^*H1047R^*; Arid1a*^*fl/fl*^ mice (Fig. 2c). Following positive selection, we analyzed purified populations by flow cytometry and observed no significant difference in purity between genotypes (Supplementary Fig. 3a, b). We isolated RNA from control and *LtfCre*^*0/+*^; (Gt)R26Pik3ca^*H1047R^*; Arid1a*^*fl/fl*^ mice. Purified *LtfCre*^*0/+*^; (Gt)R26Pik3ca^*H1047R^*; Arid1a*^*fl/fl*^ cells showed significantly reduced ARID1A messenger RNA (mRNA) expression (Fig. 2d). These samples were processed for RNA-seq, from which we observed 3481 differentially expressed genes between control and *LtfCre*^*0/+*^; (Gt)R26Pik3ca^*H1047R^*; Arid1a*^*fl/fl*^ (FDR < 0.05) (Supplementary Fig. 3c). Using stringent criteria (FDR < 10^−5^, twofold change), we identified a gene signature of 517 differentially expressed genes (Supplementary Fig. 3d). We found overlap between *LtfCre*^*0/+*^; (Gt)R26Pik3ca^*H1047R^*; Arid1a*^*fl/fl*^, *LtfCre*^*0/+*^; (Gt)R26Pik3ca^*H1047R^*; Arid1a*^*fl/+*^ and *LtfCre*^*0/+*^; (Gt)R26Pik3ca^*H1047R^; *Arid1a*^*fl/V1068G*^, including 963 genes differentially expressed in all genotypes relative to control (Supplementary Fig. 3e–g).

We performed Gene Set Enrichment Analyses (GSEA) on differentially expressed genes (FDR < 0.05) in *LtfCre*^*0/+*^; (Gt)R26Pik3ca^*H1047R^*; Arid1a*^*fl/fl*^ endometrial epithelial cells and identified EMT as the top dysregulated pathway using hallmark pathway enrichment (Fig. 2e). Mesenchymal-marker overexpression in EC correlates with poor prognosis^36^, which is consistent with several Gene Ontology (GO) pathways related to cell motility, migration and adhesion that were identified (Fig. 2f), further suggesting EMT as a key dysregulated pathway in the *LtfCre*^*0/+*^; (Gt)R26Pik3ca^*H1047R^*; Arid1a*^*fl/fl*^ endometrial epithelium. Recently, Mak et al.^37^ identified a patient-derived EMT signature of 77 genes across 11 cancer types. This gene signature was significantly enriched by GSEA in *LtfCre*^*0/+*^; (Gt)R26Pik3ca^*H1047R^*; Arid1a*^*fl/fl*^ vs. control, and in ARID1A mutant UCEC patients vs. ARID1A wild-type patients (NES = 1.72 and 1.88, respectively) (Fig. 2g), and contained 33 genes that were differentially expressed in mutant mouse endometrial cells (Fig. 2h).

EMT is characterized by the loss of cell adherens junctions, tight junctions and apical-basal polarity^38^. In *LtfCre*^*0/+*^*; Arid1a*^*fl/fl*^ mice, we observed reduced CLDN10 and tight junction protein-1 (ZO-1) expression by immunofluorescence (IF), while expression of ICAM-1 was induced, indicating impaired tight junctions (Supplementary Fig. 4a–d). ZO-1 expression was partially restored in *LtfCre*^*0/+*^; (Gt)R26Pik3ca^*H1047R^*; Arid1a*^*fl/fl*^ (Supplementary Fig. 4a). *LtfCre*^*0/+*^*; Arid1a*^*fl/fl*^ endometrium has high expression of Cleaved Caspase-3 (CASP3), indicating increased apoptosis in the absence of PIK3CA^H1047R^ (Supplementary Fig. 4e). Expression of mesenchymal-marker VIM (Vimentin) and EMT transcription factor SNAI2 (Slug) were observed in both *LtfCre*^*0/+*^*; Arid1a*^*fl/fl*^ and *LtfCre*^*0/+*^; (Gt)R26Pik3ca^*H1047R^*; Arid1a*^*fl/fl*^ mutant endometrial epithelium, indicating a shift towards a mesenchymal phenotype (Supplementary Fig. 4f, g). CDH1 (E-Cadherin) mislocalization was observed in mutant endometrial epithelium, suggesting alterations in epithelial adherens junctions (Supplementary Fig. 4h). These data suggest that the EMT phenotype observed in *LtfCre*^*0/+*^; (Gt)R26Pik3ca^*H1047R^*; Arid1a*^*fl/fl*^ endometrial epithelium are driven primarily by ARID1A loss.

### Mouse gene signature identifies invasive patient population

**Fig. 3:**
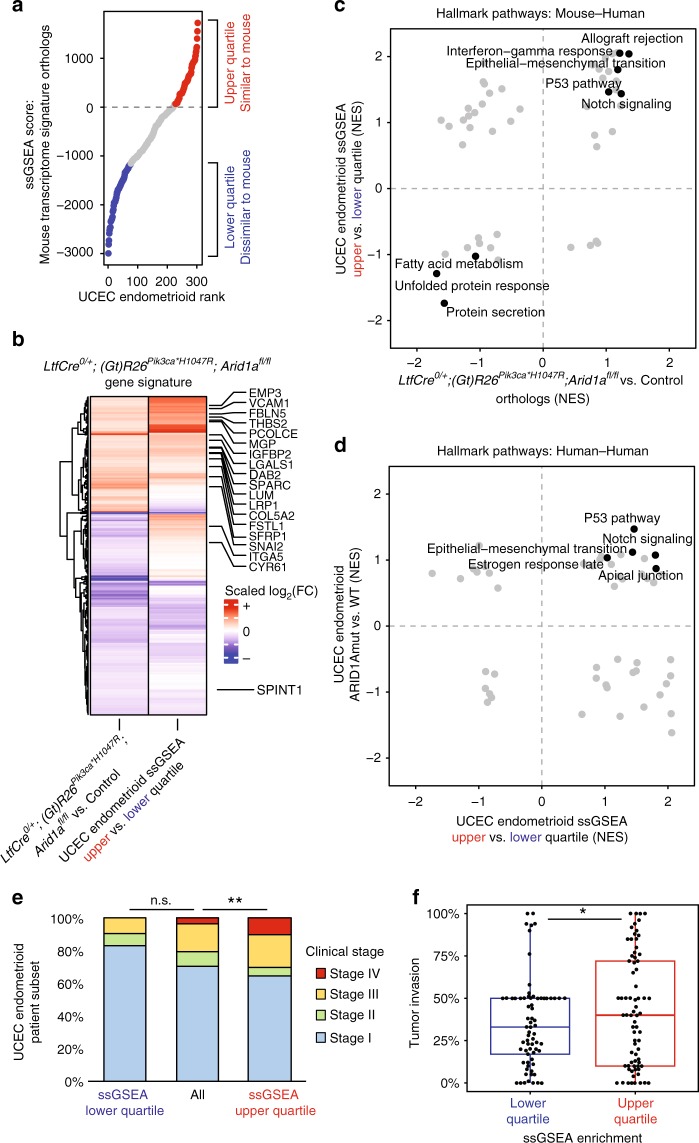
*LtfCre*^*0/+*^; (Gt)R26Pik3ca^*H1047R^*; Arid1a*^*fl/fl*^ gene signature correlates with invasive patient gene expression. **a** Distribution of TCGA-UCEC endometrioid patient tumors relative to ssGSEA score for human orthologs of *LtfCre*^*0/+*^; (Gt)R26Pik3ca^*H1047R^*; Arid1a*^*fl/fl*^ gene signature. **b** Clustered comparison of scaled fold-change values for signature genes between *LtfCre*^*0/+*^; (Gt)R26Pik3ca^*H1047R^*; Arid1a*^*fl/fl*^ vs. control mice and upper vs. lower quartile of UCEC endometrioid patients. EMT genes from Hallmark pathway and Mak and Tong pan-cancer gene signature are identified. **c** Scatter plot of Hallmark pathway GSEA Normalized Enrichment Scores (NES) from *LtfCre*^*0/+*^; (Gt)R26Pik3ca^*H1047R^*; Arid1a*^*fl/fl*^ vs. control (human orthologs) and upper quartile of UCEC endometrioid patients vs. lower quartile. **d** Scatter plot of Hallmark pathway GSEA NES from upper quartile of UCEC endometrioid patients vs. lower quartile and UCEC endometrioid ARID1A^mut^ (frameshift/truncating alterations) vs. ARID1A^wt^. **e** Upper quartile ssGSEA-enriched UCEC endometrioid patients present with higher stage disease relative to all patients (*p* < 0.01, Chi-squared). **f** Upper quartile ssGSEA-enriched UCEC endometrioid patients have more invasive tumors relative to lower quartile patients (*p* < 0.05, unpaired Mann–Whitney *U*, one-tailed). Box-and-whiskers plotted in the style of Tukey without outliers

We next wanted to determine if *LtfCre*^*0/+*^; (Gt)R26Pik3ca^*H1047R^*; Arid1a*^*fl/fl*^ gene expression patterns resembled human disease. We utilized mutation and RNA-seq expression data from the TCGA-UCEC dataset with single-sample GSEA (ssGSEA) to rank UCEC patient endometrioid tumors with gene expression patterns similar to our mouse model. We segregated the upper (similar to mouse) and lower (dissimilar to mouse) quartiles of patients based on human orthologs of our gene signature (Fig. 3a). Upper quartile UCEC patients display concordant expression changes for 74% of genes within the *LtfCre*^*0/+*^; (Gt)R26Pik3ca^*H1047R^*; Arid1a*^*fl/fl*^ gene signature relative to lower quartile patients (Fig. 3b). Upper quartile patients show upregulation of EMT, Interferon gamma (IFNγ), Notch and P53 signaling pathways, and downregulation of the unfolded protein response (UPR) (Fig. 3c). We confirmed downregulation of GRP94 and GRP78, two proteins critical to the UPR, in the *LtfCre*^*0/+*^; (Gt)R26Pik3ca^*H1047R^*; Arid1a*^*fl/fl*^ endometrial epithelium in vivo by IHC and IF (Supplementary Fig. 2b, c). When comparing ARID1A mutant and wild-type UCEC patients, we also identified upregulation of the EMT pathway (Fig. 3d).

Clinical staging of endometrial cancer is determined by invasion into surrounding tissue, including the myometrium, cervix, vagina, bladder, and distant metastasis^2^. Upper quartile patients were diagnosed with advanced clinical stage relative to all UCEC patients, with significantly more stage III and stage IV patients (*p* < 0.01, Chi-squared) (Fig. 3e). Furthermore, upper quartile patients had significantly more invasion than lower quartile patients (*p* < 0.05, unpaired Mann–Whitney *U*, two-tailed) (Fig. 3f). These data suggest that endometrial cells from *LtfCre*^*0/+*^; (Gt)R26Pik3ca^*H1047R^*; Arid1a*^*fl/fl*^ mice are representative of UCEC patients with advanced stage, invasive tumors.

### ARID1A loss increases promoter accessibility in vivo

**Fig. 4:**
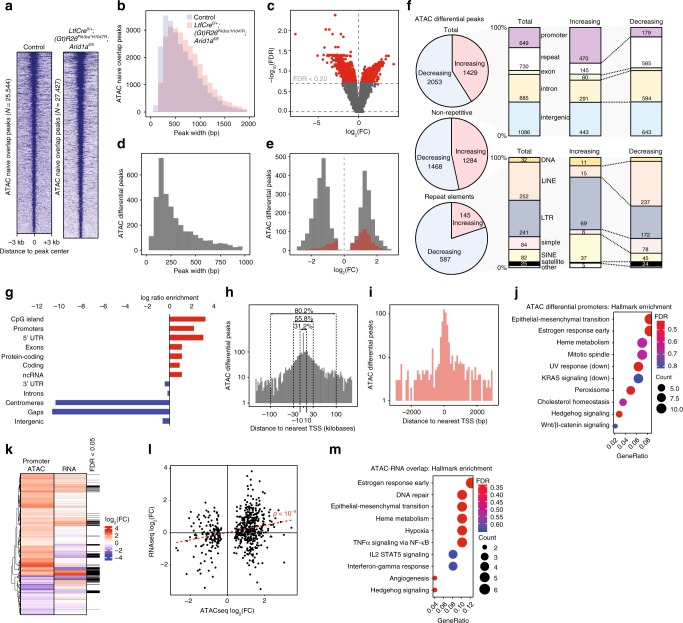
ATAC-seq analysis of differentially accessible chromatin in *LtfCre*^*0/+*^; (Gt)R26Pik3ca^*H1047R^*; Arid1a*^*fl/fl*^ endometrial epithelium. **a** ATAC-seq read density heatmap from naive overlapping peaks of control and *LtfCre*^*0/+*^; (Gt)R26Pik3ca^*H1047R^*; Arid1a*^*fl/fl*^ EPCAM-positive cells, ranked by total intensity. Reads are centered on the middle of the accessible peak ±3 kb. Control (*N* = 2, pooled groups of six mice) and *LtfCre*^*0/+*^; (Gt)R26Pik3ca^*H1047R^*; Arid1a*^*fl/fl*^ (*N* = 2, single mice). **b** Peak width distributions of control and *LtfCre*^*0/+*^; (Gt)R26Pik3ca^*H1047R^*; Arid1a*^*fl/fl*^ ATAC-seq peaks, which are significantly different (*p* < 10^−15^, unpaired Mann–Whitney *U*, two-tailed). **c** Volcano plot for differential accessibility of ATAC-seq peaks between control and *LtfCre*^*0/+*^; (Gt)R26Pik3ca^*H1047R^*; Arid1a*^*fl/fl*^ cells. Red points represent significant peaks (FDR < 0.20). **d** Peak width distribution of differentially accessible peaks. **e** Magnitude distribution of differentially accessible peaks separated by total peaks (gray) and promoter peaks (red, within 3 kb of TSS). **f** Detailed peak annotation of increasing and decreasing differentially accessible regions for total, non-repetitive and repetitive peaks based on genome annotation. **g** Enrichment for significant genomic features among differentially accessible peaks, ranked by *p*-value. Enrichment ratio is calculated by bp of feature in ATAC peak set compared to background genome. **h** Histogram of all differential ATAC peaks depicting distance to nearest TSS. Percent of peaks found within + /−10, 30, or 100 kb of the TSS are shown. **i** Histogram of differential ATAC promoter peaks depicting distance to nearest TSS. **j** mSigDb Hallmark pathway enrichment of genes with differentially accessible promoter peaks. **k** Differentially accessible promoter peak clustering based on direction and magnitude of change in gene expression and promoter accessibility. Black bars indicate significant differential gene expression by RNA-seq (FDR < 0.05). **l** Scatter plot depicting the relationship between direction and magnitude of change in accessibility and gene expression for differential promoter peaks. Accessibility and expression were significantly correlated (*r*_s_ = 0.26, *p* < 10^−9^, Spearman). **m** mSigDb Hallmark pathway enrichment of overlapping differentially accessible promoters and differentially expressed genes

To gain insight into chromatin accessibility alterations that may drive the observed gene expression changes, we performed ATAC-seq (Assay for Transposase-Accessible Chromatin)^39^ on anti-EPCAM-purified cells. In general, the peaks were broader in *LtfCre*^*0/+*^; (Gt)R26Pik3ca^*H1047R^*; Arid1a*^*fl/fl*^ cells compared to cells from control mice (*p* < 10^−15^, unpaired Mann–Whitney *U*, two-tailed), potentially indicating greater chromatin accessibility in mutant cells (Fig. 4a, b). Among differentially accessible peaks (FDR < 0.20), 2053 showed decreased accessibility in *LtfCre*^*0/+*^; (Gt)R26Pik3ca^*H1047R^*; Arid1a*^*fl/fl*^ mice, while 1429 showed increased accessibility, suggesting a global trend toward decreasing accessibility (Fig. 4c). Primarily, differentially accessible peaks represented mononucleosome fragments (Fig. 4d). Despite the trend toward decreased accessibility, among promoters (defined as regions ±3 kb to transcription start sites or TSS) we observed significant increases in accessibility (*p* < 10^−72^, Chi-squared) (Fig. 4e), with 470 promoter peaks increasing in accessibility and 179 decreasing (Fig. 4f). Genomic repeat elements trended toward decreased accessibility (80% decreasing), accounting for a global trend toward decreasing accessibility (Fig. 4f). Among peaks with increased accessibility, CpG islands, promoters and 5′ UTR were the top enriched genomic features (Fig. 4g). Differentially accessible peaks, including promoter peaks, were generally located proximal to TSS, with 31.2% of all peaks located within 10 kb of a TSS (Fig. 4h, i). We also performed ATAC-seq on EPCAM-purified cells from *LtfCre*^*0/+*^; (Gt)R26Pik3ca^*H1047R^*; Arid1a*^*fl/+*^ and *LtfCre*^*0/+*^; (Gt)R26Pik3ca^*H1047R^; *Arid1a*^*fl/V1068G*^, and observed enrichment for differential accessibility among promoters (*p* < 10^−500^) (Supplementary Fig. 3h–p).

Among genes with differentially accessible promoter peaks, EMT appeared as the top enriched pathway (Fig. 4j). We identified significant overlap between differentially accessible promoters and differentially expressed genes (*p* < 10^−8^, hypergeometric enrichment) (Fig. 4k). Chromatin accessibility was positively correlated with gene expression (*p* < 10^−9^, Spearman) (Fig. 4l). Among these genes, EMT again appeared as a top affected pathway by enrichment analysis (Fig. 4m). Altogether, these data demonstrate that endometrial ARID1A loss and PI3K activation results in increased accessibility at gene promoters and differential accessibility of EMT pathway genes.

### ARID1A functionally binds gene promoters

**Fig. 5:**
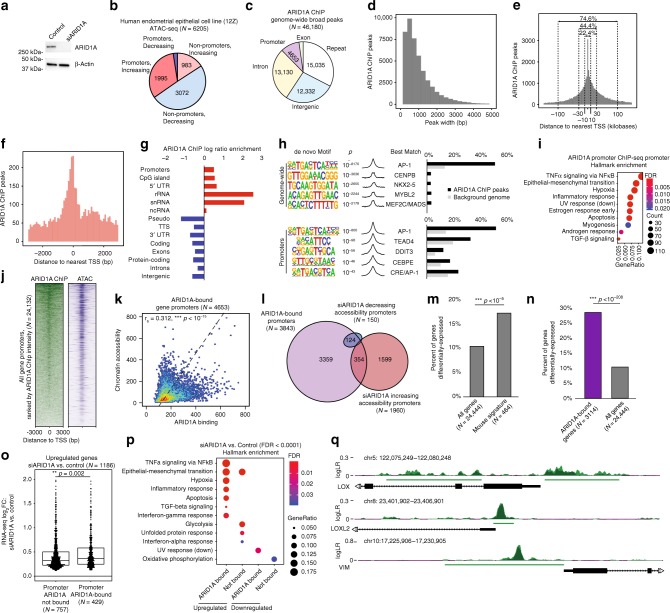
ARID1A binding is associated with accessibility and differential gene expression driven by ARID1A loss in human endometrial epithelial cell line. **a** Western blot of ARID1A expression in siRNA-treated 12Z cells. β-Actin was used as endogenous control. **b** Annotation of differentially accessible ATAC peaks (FDR < 0.05) from 12Z siARID1A, separated into fractions by directionality and promoter vs. non-promoter. Significant association (*p* < 10^−500^, Chi-squared) between increasing accessibility and promoter status. **c** Annotation of ARID1A ChIP peaks in wild-type 12Z cells. **d** Peak width distribution of ChIP peaks. **e** Histogram of all ChIP peaks depicting distance to nearest TSS. Percent of peaks found within + /−10, 30, or 100 kb of the TSS are shown. **f** Histogram of ChIP promoter peaks depicting distance to nearest TSS. **g** Enrichment for significant genomic features among ChIP peaks, ranked by *p*-value. Enrichment ratio is calculated by bp of feature in ChIP peak set compared to background genome. **h** de novo Motif enrichment of ChIP peaks genome-wide and at promoters. **i** mSigDb Hallmark pathway enrichment of genes with ChIP promoter peaks. **j** Read density heatmap of ARID1A ChIP-seq and ATAC-seq (control) at all gene promoters (*N* = 24,132), ranked by signal intensity for ARID1A ChIP-seq. **k** Scatter plot depicting correlation between ARID1A binding and chromatin accessibility (*r*_s_ = 0.312, *p* < 10^–15^, Spearman). **l** Proportional Euler diagram of overlap between ARID1A binding, decreasing and increasing chromatin accessibility at promoters. **m** Enrichment for *LtfCre*^*0/+*^; (Gt)R26Pik3ca^*H1047R^*; Arid1a*^*fl/fl*^ gene signature among 12Z siARID1A differentially expressed genes (*p* < 10^−5^, hypergeometric enrichment). **n** Enrichment of ARID1A binding at 12Z siARID1A differentially expressed genes (*p* < 10^−208^, hypergeometric enrichment). **o** Fold-change in gene expression of siARID1A upregulated genes, segregated based on ARID1A promoter-binding status (*p* *=* 0.002, unpaired Mann–Whitney *U*, two-tailed). Box-and-whiskers plotted in the style of Tukey without outliers. **p** mSigDb Hallmark pathway enrichment of 12Z siARID1A differentially expressed genes (FDR < 0.0001). **q** Example browser tracks for ARID1A binding profile. Signal is displayed as log likelihood ratio (logLR). Single replicate signal is represented in light green, overlapping signal is represented in dark green. Green bars represent peaks called

To explore the role of ARID1A loss alone in the regulation of endometrial epithelial chromatin accessibility, we utilized an immortalized human endometrial epithelial cell line, 12Z^40^. Transfection of 12Z cells with short-interfering RNAs (siRNAs) targeting ARID1A (siARID1A) reduced ARID1A protein expression relative to cells transfected with non-targeting control (siNONtg) (Fig. 5a). Next, we performed ATAC-seq on siARID1A transfected 12Z cells (Supplementary Fig. 5a–d). ARID1A loss led to a trend toward decreasing chromatin accessibility genome-wide, while chromatin accessibility was significantly increased at promoters (*p* < 10^−500^, Chi-squared) (Fig. 5b). These results recapitulate our findings in vivo, suggesting differential chromatin changes in vivo are driven by ARID1A loss alone.

In order to profile sites of genome-wide ARID1A occupancy, we performed ARID1A ChIP-seq in 12Z cells. The specificity of the ARID1A ChIP-seq antibody used was validated by co-immunoprecipitation (co-IP) and mass spectrometry (Supplementary Fig. 5e, f). We identified 46,180 unique sites of ARID1A genome-wide occupancy (Fig. 5c). The majority of ARID1A ChIP-seq peaks were less than 1000 bp in width (Fig. 5d) and generally were proximal to TSS, with roughly one-quarter of all peaks being within 10 kb of the TSS (Fig. 5e, f). ARID1A binding was significantly enriched at promoters (Fig. 5g). Among ARID1A-bound sites, we observed an enrichment of the AP-1 motif, both genome-wide (*p* < 10^−8170^) and at promoters (*p* < 10^−800^). ARID1A has been shown to regulate chromatin accessibility at AP-1 motifs^41,42^, and we also observed an enrichment for the AP-1 motif at sites of differential accessibility in vivo and in vitro (Supplementary Fig. 5g), suggesting ARID1A regulation of chromatin at AP-1 sites.

ARID1A-bound promoters were enriched for EMT hallmark genes (Fig. 5i). We also observed significant overlap between ARID1A binding and sites of accessible chromatin, which were positively correlated (*p* < 10^−15^, Spearman) (Fig. 5j, k). Among differentially accessible promoters, ARID1A was bound to 354 promoters, which increased in accessibility, and 124 promoters, which decreased in accessibility upon ARID1A loss (Fig. 5l).

To further explore the relationship between ARID1A binding and gene expression, we performed RNA-seq on siNONtg and siARID1A treated 12Z cells. Differentially expressed genes (FDR < 0.0001) were significantly enriched for the *LtfCre*^*0/+*^; (Gt)R26Pik3ca^*H1047R^*; Arid1a*^*fl/fl*^ gene signature (*p* < 10^−5^, hypergeometric enrichment) (Fig. 5m). ARID1A promoter binding was significantly enriched in differentially expressed genes with ARID1A knockdown (*p* < 10^−208^, hypergeometric enrichment) (Fig. 5n). While ARID1A promoter binding was evenly distributed among upregulated and downregulated genes (Supplementary Fig. 5h), we observed a higher degree of gene upregulation following ARID1A loss among genes with ARID1A binding at the promoter (*p* = 0.002, unpaired Mann–Whitney *U*, two-tailed) (Fig. 5o). ARID1A bound, upregulated genes are enriched for EMT pathways (Fig. 5p). ARID1A binding is observed in the promoters of mesenchymal identity genes (Fig. 5q). These data support a mechanistic role for ARID1A in the suppression of mesenchymal gene transcription.

### ARID1A loss promotes mesenchymal phenotype

**Fig. 6:**
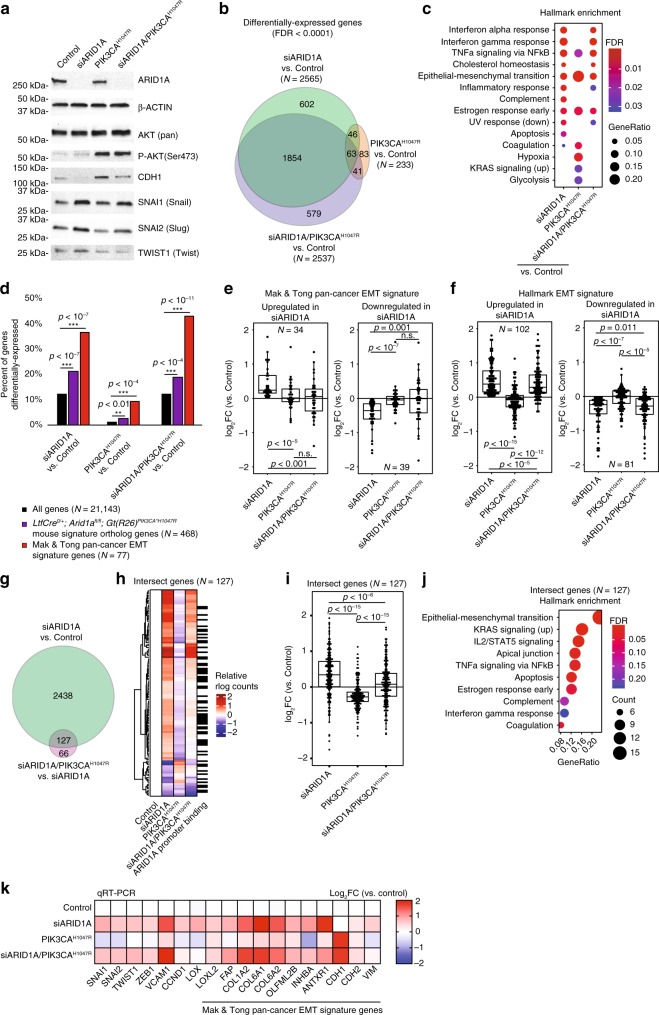
PIK3CA^H1047R^ antagonizes ARID1A loss-induced mesenchymal phenotypes. **a** Western blot of ARID1A, β-Actin, AKT, P-AKT, CDH1, SNAI1, SNAI2, and TWIST1 following co-transfection of siNONtg and empty vector (control), siARID1A and empty vector (siARID1A), siNONtg and pPIK3CA^H1047R^ (PIK3CA^H1047R^), or siARID1A and pPIK3CA^H1047R^ (siARID1A/PIK3CA^H1047R^). **b** Proportional Euler diagram displaying differentially expressed genes (FDR < 0.0001) from siARID1A, PIK3CA^H1047R^, and siARID1A/PIK3CA^H1047R^ relative to control. **c** mSigDb Hallmark pathway enrichment for siARID1A, PIK3CA^H1047R^, and siARID1A/PIK3CA^H1047R^ differentially expressed genes. **d** Enrichment for *LtfCre*^*0/+*^; (Gt)R26Pik3ca^*H1047R^*; Arid1a*^*fl/fl*^ mouse signature ortholog genes and Mak et al. pan-cancer gene signature within differentially expressed genes from siARID1A, PIK3CA^H1047R^, and siARID1A/PIK3CA^H1047R^ relative to control. **e**, **f** Fold-change values of experimental groups relative to control for genes in the Mak and Tong pan-cancer EMT signature (**e**) and the Hallmark EMT signature (**f**), separated based on direction of gene expression change in siARID1A. Statistic represented is paired Mann–Whitney *U* (two-tailed). Box-and-whiskers plotted in the style of Tukey without outliers. **g** Intersection between siARID1A differentially expressed genes relative to control and siARID1A/PIK3CA^H1047R^ relative to siARID1A. **h** Heat map detailing relative expression of intersecting genes (*N* = 127) (Fig. 6g) in control, siARID1A, PIK3CA^H1047R^, and siARID1A/PIK3CA^H1047R^, and ARID1A promoter binding. These genes were enriched for ARID1A promoter binding (*p* < 10^−18^, hypergeometric enrichment). **i** Expression level of intersect genes (Fig. 6g) in siARID1A, PIK3CA^H1047R^, and siARID1A/PIK3CA^H1047R^ relative to control. Statistic represented is paired Mann–Whitney *U* (two-tailed). Box-and-whiskers plotted in the style of Tukey without outliers. **j** mSigDb Hallmark pathway enrichment for intersecting genes (*N* = 127) (Fig. 6g). **k** Changes in relative EMT gene expression upon ARID1A loss and PIK3CA^H1047R^ overexpression as measured by qRT-PCR. Data represents three biological replicates

To further interrogate the relationship between ARID1A and PIK3CA in the regulation of the EMT pathway, we again utilized the 12Z cell line. EMT is regulated by several transcription factors, including SNAI1 (Snail), SNAI2 and TWIST1 (Twist)^38^. Upon ARID1A knockdown by siRNA (siARID1A), we observed upregulation of SNAI1, SNAI2, and TWIST1 protein expression (Fig. 6a). Transfection with PIK3CA^H1047R^ expression plasmid (pPIK3CA^H1047R^) led to AKT/mTOR pathway activation, as indicated by phosphorylation of AKT at serine 473 (P-AKT Ser473) (Fig. 6a). In cells transfected with both siARID1A and pPIK3CA^H1047R^, we observed decreased induction TWIST1 (Fig. 6a). Expression of SNAI1 and SNAI2 was not affected by pPIK3CA^H1047R^ (Fig. 6a). Moreover, pPIK3CA^H1047R^ induced CDH1 expression (Fig. 6a) and partially rescued the CDH1 downregulation observed in cells transfected with only siARID1A.

We next performed RNA-seq on cells transfected with siARID1A, pPIK3CA^H1047R^, or both. We found that while ARID1A loss resulted in differential gene expression of 2565 genes, PIK3CA^H1047R^ expression resulted in differential gene expression of only 233 genes (FDR < 0.0001) (Fig. 6b). Some genes differentially expressed by PIK3CA^H1047R^ overlapped with siARID1A and siARID1A/PIK3CA^H1047R^ samples, displaying unique patterns of gene expression (Supplementary Fig. 6). Among Hallmark pathways, we observed siARID1A and PIK3CA^H1047R^ convergence on the NFκB pathway, as previously described in ovarian clear cell carcinoma^43^, and the EMT pathway (Fig. 6c). Differentially expressed genes from siARID1A, PIK3CA^H1047R^, and siARID1A/PIK3CA^H1047R^ samples compared to controls were enriched for the *LtfCre*^*0/+*^; (Gt)R26Pik3ca^*H1047R^; *Arid1a*^*fl/fl*^ gene signature and the Mak and Tong pan-cancer EMT gene signature (Fig. 6d). For genes found in the Mak and Tong signature and the hallmark EMT pathway, we identified an antagonistic relationship between siARID1A and PIK3CA^H1047R^, such that gene expression changes observed in siARID1A samples were reduced in siARID1A/PIK3CA^H1047R^ samples (Fig. 6e, f).

To further explore the antagonistic relationship between ARID1A loss and PIK3CA^H1047R^, we identified a unique group of genes at the intersection between differentially expressed genes in siARID1A relative to control and siARID1A/PIK3CA^H1047R^ relative to siARID1A (Fig. 6g). These 127 genes represent genes, which are differentially expressed by siARID1A, and further altered by the addition of PIK3CA^H1047R^. Of these genes, 47.2% were bound by ARID1A at the promoter in wild-type 12Z cells (*p* < 10^−18^, hypergeometric enrichment) (Fig. 6h). We observed significant upregulation of these genes in siARID1A samples, and downregulation in siARID1A/ PIK3CA^H1047R^ (Fig. 6h, i). These genes were enriched for the hallmark EMT pathway, which was the most significant result (Fig. 6j). The differential gene expression of EMT genes upon ARID1A loss was confirmed by quantitative reverse transcriptase (qRT)-PCR (Fig. 6k). These data provide further evidence that ARID1A loss induces a mesenchymal phenotype, which is antagonized by the PIK3CA^H1047R^ mutation, resulting in a partial EMT phenotype.

### ARID1A loss and PIK3CA^H1047R^ promote invasive phenotypes

**Fig. 7:**
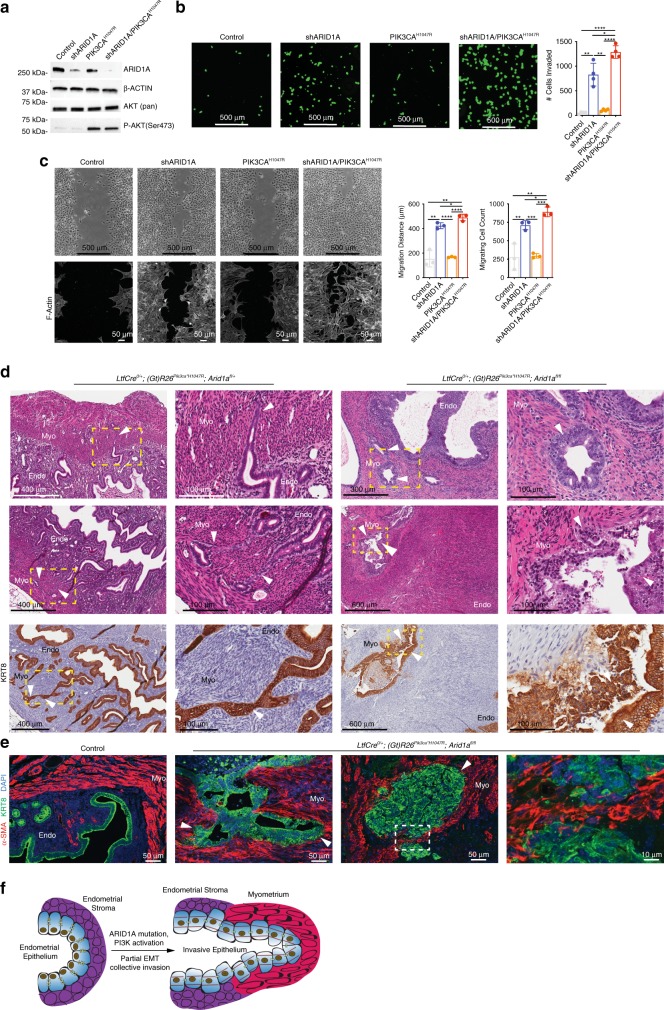
ARID1A loss and PIK3CA^H1047R^ promote myometrial invasion in vivo and migration in vitro. **a** Western blot of ARID1A, β-Actin, AKT, P-AKT, following co-transfection of shNONtg and empty vector (control), shARID1A and empty vector (shARID1A), shNONtg and pPIK3CA^H1047R^ (PIK3CA^H1047R^) or shARID1A and pPIK3CA^H1047R^ (shARID1A/PIK3CA^H1047R^). **b** Invasion assay of 12Z cells with ARID1A loss and PIK3CA^H1047R^ overexpression. Representative images of calcein AM-stained cells are and total invaded cell counts are shown (scale bar = 500 μm). Data represents four biological replicates (mean ± s.d; *p < 0.05, **p < 0.01, ****p < 0.0001, unpaired *t*-test, two-tailed). **c** Migration assay of 12Z cells with ARID1A loss and PIK3CA^H1047R^ overexpression. Upper images are representative of cells 24 h following removal of insert (scale bar = 500 μm). Lower images are maximum intensity confocal projections of cells stained with fluorescent phalloidin to label with F-actin (scale bar = 50 μm). Average Migration represents the average difference distance across each migration front from 0 to 24 h. Migrating cell counts represent number of cells in migration area after 24 h. Data represents three biological replicates (mean ± s.d; *p < 0.05, **p < 0.01, ***p < 0.001, ****p < 0.0001, unpaired *t*-test, two-tailed). **d** Myometrial invasion observed in *LtfCre*^*0/+*^*; Arid1a*^*fl/fl*^, and *LtfCre*^*0/+*^; (Gt)R26Pik3ca^*H1047R^*; Arid1a*^*fl/fl*^. H&E staining and IHC for KRT8 at 3.33–6.66 × (scale bar = 300–600 μm, as stated on figure) and x20 (scale bar = 100 μm) magnification, with x20 magnifications representing portion panel to the right surrounded by yellow box. White arrows indicate invasive endometrial epithelium. Endo, endometrium; Myo, myometrium. **e** Images of maximum intensity confocal projections of control and *LtfCre*^*0/+*^; (Gt)R26Pik3ca^*H1047R^*; Arid1a*^*fl/fl*^ endometrium sections stained with α-smooth muscle actin (α-SMA) (red), KRT8 (green) and counter-stained with DAPI (blue) (*N* ≥ 3). White arrows indicate invasive endometrial epithelium (scale bar = 50 or 10 μm, as stated on figure). **f** Diagram representation of EMT-induced invasive endometrial epithelium following ARID1A loss and PIK3CA^H1047R^ mutation

Partial EMT is associated with invasive phenotypes^38^, and EMT pathways play key roles in EC disease progression by promoting the invasion of epithelial cells into the myometrium^44^. To distinguish between the effect of ARID1A loss or PIK3CA^H1047R^ on invasive phenotypes, we co-transfected 12Z cells with a PIK3CA^H1047R^ expression plasmid and lentivirus expressing ARID1A short-hairpin RNAs (shRNAs) (shARID1A) (Fig. 7a). ARID1A knockdown induced migratory and invasive phenotypes in 12Z cells, and co-transfection with pPIK3CA^H1047R^ significantly enhanced migration and invasion (Fig. 7b, c). Cells treated with shARID1A displayed increased expression of F-actin (Fig. 7c). These results suggest that the co-mutation of ARID1A and PIK3CA in the endometrial epithelium promotes an invasive phenotype.

In vivo, we observed a requirement for both ARID1A loss and PI3K activation for invasive phenotypes. In *LtfCre*^*0/+*^; (Gt)R26Pik3ca^*H1047R^*; Arid1a*^*fl/+*^ and *LtfCre*^*0/+*^; (Gt)R26Pik3ca^*H1047R^*; Arid1a*^*fl/fl*^ mice, we observed invasion of endometrial epithelium into the myometrium (Fig. 7d). KRT8-positive epithelial cells migrated outside of the endometrium, invading α-smooth muscle actin (α-SMA)-positive myometrial cells and formed tumors (Fig. 7e). Invading epithelial cells contained a narrow leading edge and strand-like morphology, suggesting a collective migration of cells^45^. Some invasive sites formed well-differentiated adenomas (Fig. 7d), while others were poorly differentiated clusters of tumor cells (Fig. 7d, e). Invasive KRT8-positive epithelial glands were observed in direct contact with myometrial cells, often appearing as strands of epithelial cells trailing through the myometrial layers (Fig. 7d). These results suggest that ARID1A loss and PIK3CA^H1047R^ expression in the endometrial epithelium results in a partial EMT phenotype, promoting lesion formation and myometrial invasion (Fig. 7f).

## Discussion

In this study, we found that ARID1A functions as a haploinsufficient tumor suppressor in the endometrial epithelium. *LtfCre*^*0/+*^; (Gt)R26Pik3ca^*H1047R^; *Arid1a*^*fl/+*^ is sufficient to drive tumorigenesis and is nearly identical to *LtfCre*^*0/+*^; (Gt)R26Pik3ca^*H1047R^*; Arid1a*^*fl/fl*^ with respect to tumor burden, survival, gene expression, and chromatin accessibility changes. This is consistent with the spectrum of single-hit ARID1A mutations observed in EC, in which a majority of patients have only a single ARID1A nonsense mutation. Previous studies suggested ARID1A functions as a haploinsufficient tumor suppressor^31^ in ovarian^10,11^, breast^46^, gastric^47^, and liver cancer^48^. ARID1A expression or mutation may not predict disease status, as single-hit mutations or epigenetic silencing may be sufficient for ARID1A-dependent changes in gene expression or transformation. Additionally, heterozygous loss of ARID1A may promote metastasis at late stages of the tumor progression, as observed in liver cancer^49^. ARID1A levels may be regulated throughout the menstrual cycle and mediate dissociation of decidua from the uterus. In this case, ARID1A heterozygosity may suffice for oncogenesis during points of low ARID1A expression, which may account for the ARID1A genetic differences observed between the present mouse model and epithelial ovarian cancer models^29,30^. This would explain the high ARID1A mutation rates in EC.

Previously, Raab et al.^50^ identified ARID1A binding preferentially at promoters in HepG2 liver cancer cells. In the present study, we show ARID1A enrichment at promoters, which was significantly correlated with chromatin accessibility. We observed increased accessibility at promoters upon ARID1A loss in human endometrial epithelial cells and, in vivo, in sorted mouse endometrial epithelial cells. Among direct ARID1A target genes, we also observed significant correlations between increasing promoter accessibility and increasing transcription of mesenchymal genes upon ARID1A loss. In addition, we observed greater activation of ARID1A target genes following ARID1A loss, as compared to ARID1A non-target genes. These data suggest ARID1A-containing SWI/SNF complexes maintain endometrial epithelial cell identity by repressing genes required for transdifferentiation of epithelial cells into mesenchyme. ARID1A may promote endometrial plasticity by limiting the differentiation capacity of the epithelial cells. Repressive nucleosome positioning by ARID1A-containing SWI/SNF complexes may provide a barrier to transcriptional activation, as has been observed at the HIV LTR^51^.

The data presented here demonstrate a cell-autonomous role for ARID1A in the preservation of endometrial epithelial cell identity and EMT regulation. In addition, we show *LtfCre*^*0/+*^; (Gt)R26Pik3ca^*H1047R^*; Arid1a*^*fl/fl*^ cells gain VIM and ICAM-1 and invade the myometrium, but retain CDH1, EPCAM and KRT8 expression, suggesting an incomplete EMT phenotype^38^. VIM expression is upregulated in epithelial tumors of uterine corpus origin, but not epithelial tumors of ovarian origin^52^. ICAM-1 is expressed in migratory EC^53^, and is linked to increased peritoneal adhesion in endometriosis^54^. VIM and ICAM-1 may serve as markers of ARID1A-negative tumors of endometrial origin.

Partial or incomplete EMT is associated with invasive phenotypes in various cancers^38,45^. In EC, EMT is thought to play a role in myometrial invasion^44^. In this study, we found that ARID1A loss and PI3K activation in endometrial epithelium leads to enhanced migration and invasion in vitro and myometrial invasion in vivo, reflecting the myometrial invasion phenotypes observed clinically. Myometrial invasion in EC correlates with distal metastases, disease recurrence, and adenomyosis^55–57^. EC patients with gene expression signatures most similar to *LtfCre*^*0/+*^; (Gt)R26Pik3ca^*H1047R^*; Arid1a*^*fl/fl*^ had greater tumor invasion and higher tumor grade. The collective migration of mutant endometrial epithelium undergoing partial EMT may enhance the invasive properties of EC, permitting myometrial invasion.

The retention of some epithelial characteristics upon PIK3CA^H1047R^ expression may facilitate the establishment of epithelial tumors^58^. Epithelial transdifferentiation is a proposed mechanism by which normal epithelia convert into abnormal epithelia without undergoing an mesenchymal cell intermediate^59^. PIK3CA mutation is an early event in atypical hyperplasia^24^, whereas loss of ARID1A immunoreactivity correlates with malignant transformation in endometrial cancer^15^. A recent study identified PIK3CA as being commonly mutated in endometrial glands, often without transformation, suggesting PIK3CA mutation as an early event, with ARID1A mutation coming later in the progression of endometriosis^17^. ARID1A mutations have previously been implicated in invasion during metastasis^49,60–62^. In the *LtfCre*^*0/+*^; (Gt)R26Pik3ca^*H1047R^*; Arid1a*^*fl/fl*^ endometrial epithelium, PI3K activation may partially suppress the full acquisition of mesenchymal phenotypes upon ARID1A loss, resulting in an abnormal epithelial state with invasive properties. PI3K activation may also allow cells to bypass the endometrial epithelial cell apoptosis observed in *LtfCre*^*0/+*^*; Arid1a*^*fl/fl*^ mice. This may be another reason why ARID1A mutations are commonly observed alongside activating PI3K mutations in neoplasms originating from the endometrial epithelium.

The partial EMT phenotype may increase the invasive potential of the endometrium. The expression of EMT factors is increased at the myoinvasive front of ECs^44^, suggesting collective migration rather than single cell metastasis^63^. Endometriotic lesions retain CDH1 expression^64^, suggesting collective migration rather than metastasis via a single cell^63^. Within primary tumors, adjacent cells may differentiate into different intermediate stages along the EMT-spectrum due to differing stimulus within the tumor microenvironment, including surrounding stroma^38^. Invasive, mesenchymal-like cells may lead the way for cohorts of epithelial cells with which they retain some cell-cell junctions^63^. Upon arrival to metastatic sites, lack of stromal signals present at the site of origin may allow for epithelial gland formation^58^. This may explain the formation of endometrial glands outside of the endometrium derived from cells with mesenchymal-like invasiveness.

EC survival rates are high if the disease is detected at an early stage when the tumors are still confined to the endometrium. Myometrial invasion or tumor dissemination to other sites in the body correlates with poor survival. The notion that collective epithelial invasion promotes EC metastasis may lead to therapeutic options for patients with disseminated disease. The identification of pathways involved in the collective invasion may lead to the development of anti-metastatic drugs.

## Methods

### Mice

All mice were maintained on an outbred genetic background using CD-1 mice (Charles River). (Gt)R26Pik3ca^*H1047R^ and *LtfCre* (*Tg(Ltf-iCre)14Mmul)* alleles were purchased from The Jackson Laboratory and identified by PCR using published methods^32,33^. *Arid1a*^*fl*^ and *Arid1a*^*V1068G*^ alleles were distinguished by PCR^20,30^. For detection of *Arid1a*^*V1068G*^ allele, PCR product was treated with HincII at 37 °C for 1 h. Genotyping primers are listed in Supplementary Table 1. Uncropped genotyping gels can be found in Supplementary Fig. 7. Endpoints were vaginal bleeding, severe abdominal distension, and signs of severe illness, such as dehydration, hunching, jaundice, ruffled fur, signs of infection, or non-responsiveness. Sample sizes within each genotype were chosen based on the proportions of animals with vaginal bleeding between each experimental group or a Kaplan–Meyer log-rank test for survival differences. For weight measurements, uteri were collected at time of sacrifice and placed immediately into neutral-buffered formalin at 4 °C. After 24 h, tissues were washed with phosphate-buffered saline (PBS) and 50% EtOH, placed in 70% EtOH, and then weighed. Mice were housed at the Van Andel Research Institute Animal Facility and the Michigan State University Grand Rapids Research Center in accordance with protocols approved by Michigan State University.

### Cell lines

12Z immortalized human endometrial epithelial cells were provided by the laboratory of Asgi Fazleabas^40^. 12Z cells were maintained in Dulbecco's Modified Eagle Media (DMEM)/F12 media supplemented with 10% fetal bovine serum (FBS), 1% L-glutamine and 1% penicillin/streptomycin (P/S). Lenti-X^TM^ 293T (Clontech, Cat# 632180, CVCL_0063) cells were maintained in DMEM + 110 mg/L Sodium Pyruvate (Gibco) supplemented with 10% FBS, 1% l-glutamine, 1% P/S. Cell line validation for the 12Z cell line was performed by IDEXX BioResearch: the 12Z cell line has a unique profile not found in the current public databases. The 12Z and Lenti-X 293T cell lines tested negative for mycoplasma contamination. Testing was performed using the Mycoplasma PCR Detection Kit (Applied Biological Materials). No commonly misidentified cell lines were used in this study.

### Histology and immunohistochemistry

For indirect immunohistochemistry (IHC), 10% neutral-buffered formalin (NBF)-fixed paraffin sections were processed for heat-based antigen unmasking in 10 mM sodium citrate [pH 6.0]. Sections were incubated with antibodies at the following dilutions: 1:200 ARID1A (D2A8U) (12354, Cell Signaling); 1:400 Phospho-S6 (4585, Cell Signaling); 1:100 KRT8 (TROMA1, DHSB); 1:100 EPCAM (G8.8-s, DHSB); 1:400 PGR (SAB5500165, Sigma). TROMA-I antibody was deposited to the DSHB by Brulet, P./Kemler, R. (DSHB Hybridoma Product TROMA-I). EPCAM antibody (G8.8) was deposited to the DSHB by Farr, A.G. (DSHB Hybridoma Product G8.8). Antibody details are listed in Supplementary Table 2. The following Biotin-conjugated secondary antibodies were used: donkey anti-rabbit IgG (711-065-152, Jackson Immuno-research Lab) and donkey anti-rat IgG (#705-065-153, Jackson Immuno-research Lab). Secondary antibodies were detected using VECTASTAIN Elite ABC HRP Kit (Vector). Sections for IHC were lightly counter-stained with Hematoxylin QS or Methyl Green (Vector Labs). Routine Hematoxylin and Eosin (H&E) staining of sections was performed by the Van Andel Research Institute (VARI) Histology and Pathology Core. A VARI animal pathologist reviewed histological tumor assessments.

### Immunofluorescence

For indirect immunofluorescence, tissues were fixed in 4% paraformaldehyde. Frozen samples were sectioned at 10 μm on a CM3050 S cryostat (Leica) and collected on white frosted, positive charged ultra-clear microscope slides (Denville). Frozen slides were post-fixed with 2% PFA/1 PBS, and permeabilized with 0.3% TX100 in PBS, and treated with 100 mM glycine/1x PBS [pH 7.3]. Primary antibodies were applied to slides at the following dilutions: 1:200 ARID1A (D2A8U) (12354, Cell Signaling); 1:100 KRT8 (TROMA1, DHSB); 1:100 EPCAM (G8.8-s, DHSB); 1:50 ZO-1 (61–7300, ThermoFisher); 1:200 CDH1 (3195, Cell Signaling); 1:100 CLDN10 (38–8400, ThermoFisher); 1:100 VIM (5741, Cell Signaling); 1:400 PGR (SAB5500165, Sigma); 1:200 ERα (ab32063, abcam); 1:2000 SMA (Sigma, C618); 1:100 SNAI2 (9585, Cell Signaling); 1:40 ICAM-1 (AF796-SP, R&D Systems). Secondary antibodies used were: 1:500 donkey anti-rabbit IgG, alexa fluor 555-conjugated antibody (#A-31572, ThermoFisher); 1:500 goat anti-rabbit IgG, alexa fluor 555-conjugated antibody (#A-21428, ThermoFisher); 1:500 goat anti-rat IgG, alexa fluor 647-conjugated antibody (A-21247, ThermoFisher); 1:250 donkey anti-rat IgG, alexa fluor 647-conjugated antibody (712-605-153, Jackson Immuno-Research Lab); 1:250 donkey anti-goat fluor 488-conjugated antibody (705-545-147, Jackson Immuno-Research Lab). Phalloidin-iFluor 594 (1:1000, abcam) was used to stain F-actin. Auto-fluorescence was quenched using the TrueVIEW Auto-fluorescence Quenching Kit (Vector Laboratories). ProLong Gold Antifade Reagent with DAPI (8961, Cell Signaling) was used for DAPI staining.

### Microscopy and imaging

Confocal images were taken on a Nikon Eclipse T*i* inverted microscope using a Nikon C2 + confocal microscope laser scanner. Confocal immunofluorescent images are representative maximum intensity projections.

### Cell sorting

Mouse uteri were surgically removed and minced using scissors. Tissues were digested using the MACS Multi Tissue Dissociation Kit II (Miltenyi Biotec) for 80 min at 37 °C. Digested tissues were strained through a 40 μm nylon mesh (ThermoFisher). The Red Cell Lysis Buffer (Miltenyi Biotec) was used to remove red blood cells. Dead cells removed using the MACS Dead Cell Removal Kit (Miltenyi Biotec), and EPCAM-positive cells were positively selected and purified using a PE-conjugated EPCAM antibody and anti-PE MicroBeads (Miltenyi Biotec), per the manufacturers’ instructions. A BD Accuri C6 flow cytometer (BD Biosciences) was used to confirm purity of EPCAM-positive population.

### RNA isolation and qRT-PCR

The Arcturus PicoPure RNA Isolation Kit (ThemoFisher), including an on-column DNA digestion using the RNAse-free DNAse set (Qiagen), was used to purify RNA from in vivo EPCAM-sorted endometrial epithelial cells. To confirm loss of ARID1A transcript in EPCAM-positive *LtfCre*^*0/+*^; (Gt)R26Pik3ca^*H1047R^; *Arid1a*^*fl/fl*^ cells, complementary DNA (cDNA) was synthesized from RNA, and qRT-PCR was performed using Ssofast PCR master mix (Biorad) using previously described primers^20^ and the Applied Biosystems ViiA7 real-time PCR system. ARID1A expression was normalized to GAPDH. For in vitro experiments, RNA samples were collected 72 h post siRNA transfection using the Quick-RNA Miniprep Kit (Zymo Research). cDNA was synthesized from RNA, and qRT-PCR was performed using PowerUp SYBR Green Master Mix (ThermoFisher) and the Applied Biosystems ViiA7 real-time PCR system. Primer pairs for human genes are described in Supplementary Table 1.

### RNA-seq

Libraries were prepared by the Van Andel Genomics Core from 100 ng of total RNA for mouse samples, and Lexogen SIRV-set2 RNAs (Lexogen GmbH, Vienna Austria) were spiked into RNA prior to library preparation at a concentration of 1% by mass. For human samples, 500 ng of total RNA material was used as input, with no spike in. For all samples, libraries were generated using the KAPA Stranded mRNA-Seq Kit (v4.16) (Kapa Biosystems, Wilmington, MA USA). RNA was sheared to 250–300 bp and reverse transcribed. Prior to PCR amplification, cDNA fragments were ligated to Bio Scientific NEXTflex Adapters (Bio Scientific, Austin, TX, USA). Quality and quantity of the finished libraries were assessed using a combination of Agilent DNA High Sensitivity chip (Agilent Technologies, Inc.), QuantiFluor dsDNA System (Promega Corp., Madison, WI, USA), and Kapa Illumina Library Quantification qPCR assays (Kapa Biosystems). All libraries were pooled equimolarly, and single end sequencing to a minimum depth of 30 M reads per library was performed using an Illumina NextSeq 500 sequencer using a 75 bp sequencing kit (v2) (Illumina Inc., San Diego, CA, USA). Base calling was done by Illumina NextSeq Control Software (NCS) v2.0 and output of NCS was demultiplexed and converted to FastQ format with Illumina Bcl2fastq v1.9.0.

### RNA-seq analysis

Raw 75 bp reads were trimmed with *cutadapt*^65^ and *Trim Galore!* (http://www.bioinformatics.babraham.ac.uk/projects/trim_galore/) followed by quality control analysis via *FastQC*^66^. Trimmed mouse reads were aligned to mm10 genome assembly and indexed to GENCODE^67^ vM16 GFF3 annotation via *STAR*^68^ aligner with flag ‘–quantMode GeneCounts’ for feature counting, and human reads were aligned to GRCh38.p12 and indexed to GENCODE v28. For mouse libraries, Lexogen SIRVome was independently aligned and quantified for qualitative assessment of library concordance. Output gene count files were constructed into an experimental read count matrix in R. Low count genes were filtered (1 count per sample on average) prior to *DESeq2*^69,70^ count normalization and subsequent differential expression analysis. Calculated differential expression probabilities were corrected for multiple testing by independent hypothesis weighting (IHW)^71^ for downstream analysis. Differentially expressed gene thresholds were set at FDR < 0.05 for mouse data and FDR < 0.0001 for human data. All reported instances of log_2_(fold-change) data from RNA-seq are adjusted by *DESeq2* original shrinkage estimator except for TCGA-UCEC comparisons and statistical comparisons between log_2_(FC) values, which use non-adjusted values. Principal component analysis was calculated using *DESeq2* from top 500 genes by variance across samples. RNA-seq heatmaps were generated using scaled regularized-logarithm (rlog) counts for visualization, or relative to controls by subtracting mean rlog counts. *LtfCre*^*0/+*^*; (Gt)R26*^Pik3ca*H1047R^*; Arid1a*^*fl/fl*^ signature genes were defined by FDR < 10^−5^ and |log_2_(FC)| > 1.

### ATAC-seq

Libraries were prepared following previously described methods^39,72,73^. Mouse endometrial cells were isolated using methods described above. For purified mouse endometrial epithelium and 12Z cells, between 25,000 and 50,000 cells were resuspended in cold lysis buffer (10 mM Tris-HCL [pH 7.4], 10 mM NaCl, 3 mM MgCl_2_, 0.1% NP-40) and centrifuged at 500 × *g*, 4 °C for 10 min to isolate nuclei. Nuclei were treated with Tn5 Transposase for 30 min at 37 °C using the Nextera DNA Library Prep Kit (Illumina). DNA was isolated using the Qiagen MinElute Reaction Cleanup Kit. Libraries were amplified using barcoded primers for 1–8 cycles as described^39^. Libraries were purified using Kapa Pure Beads to remove primer dimers and >1000 bp fragments. Libraries were sequenced by the Van Andel Genomics Core. Quality and quantity of the finished libraries were assessed using a combination of Agilent DNA High Sensitivity chip (Agilent Technologies, Inc.), QuantiFluor dsDNA System (Promega Corp., Madison, WI, USA), and Kapa Illumina Library Quantification qPCR assays (Kapa Biosystems). All libraries were pooled equimolarly, and paired end sequencing to a minimum depth of 20 M reads per library was performed using an Illumina NextSeq 500 sequencer using a 150 bp sequencing kit (v2) (Illumina Inc., San Diego, CA, USA). Base calling was done by Illumina NextSeq Control Software (NCS) v2.0 and output of NCS was demultiplexed and converted to FastQ format with Illumina Bcl2fastq v1.9.0.

### ATAC-seq analysis

Libraries were combined across flow cells and trimmed with *cutadapt* and *Trim Galore!* followed by quality control analysis via *FastQC*. Trimmed reads were aligned to mm10 mouse reference genome via *Bowtie2*^74^ with flags ‘–very-sensitive’ and ‘-X 1000’ in concordance with the library size-selection step, and, similarly, human reads were aligned to GRCh38.p12 using the same parameters^75^. Reads were then sorted and indexed with *samtools*^76^. Mitochondrial reads were then discarded from BAMs, using Harvard ATAC-seq module *removeChrom* script (https://github.com/harvardinformatics/ATAC-seq), and subsequently filtered for only properly paired reads by *samtools view -f 3*. At this step, working library complexity was estimated by *ATACseqQC::estimateLibComplexity*^77,78^. To compensate for differing library complexities within an experimental design, we normalized by randomly subsampling libraries to a calculated fraction of the original library, as estimated by the bootstrap interpolation, via *samtools* view with flag ‘-s’ to achieve normalized library sizes. After subsampling libraries to lowest complexity, PCR duplicates were removed with *Picard MarkDuplicates* (http://broadinstitute.github.io/picard/), and reads were finally name-sorted prior to conversion to BEDPE format with *bedtools*^79^ *bamtobed* with flag ‘-bedpe’. BEDPE coordinates were then shifted 4 and 5 bp to correct for TN5 transposase integration^39^, and the standard BEDPE files were re-written to a minimal BEDPE format, as defined by *MACS2* manual, through an *awk* script. *MACS2*^80^ was used to call broad peaks from final minimal BEDPE fragment coordinates with FDR < 0.05 threshold and no control input, and the resulting peaks were repeat-masked by blacklist filtering^81^. A naive overlap peak set, as defined by ENCODE, was constructed for each biological condition by combining replicates and calling broad peaks on pooled BEDPE files followed by *intersectBed* to select for peaks of at least 50% overlap with each biological replicate.

Differential accessibility was calculated by firstly defining a more relaxed consensus peak set $p = (\mathop { \cap }\limits_{j = 1}^n e_j) \cup (\mathop { \cap }\limits_{j = 1}^n c_j)$ for any partial intersect where *e*_1_, …, *e*_n_ are *MACS2* peak sets from biological replicates of the experimental condition, and *c*_1_, …, *c*_*n*_ are peak sets from control biological replicates. This consensus peak set was used in *csaw*^82^ as coordinates for counting reads within specified windows, with additional parameters set to restrict windows to standard chromosomes and non-blacklisted regions. Windows >1 kilobase in width were filtered along with low read-abundance windows (logCPM < −3). In order to compensate for differing efficiencies of reactions between libraries, a non-linear loess-based normalization approach was employed to remove trended biases. This method was empirically determined to elicit the most conservative results as opposed to other approaches to window count normalization. *csaw* uses *edgeR*^83^ quasi-likelihood functionality to calculate differential accessibility, for which FDR thresholds were used to determine final differential peak sets (FDR < 0.20 mouse data; FDR < 0.05 human data). Finally, proximal windows within 500 bp were merged, and the most significant window statistic was used to represent the merged window.

Significant differentially accessible genomic regions were annotated by *HOMER*^84^ with a modification to *cis*-promoter classification as within 3000 bp of a canonical gene TSS, which remains consistent throughout all reported analyses. *HOMER* de novo motif enrichment and genome ontology was performed on all significant differentially accessible genomic regions. Common differential mouse ATAC/RNA genes were selected by the presence of a differentially accessible promoter ATAC peak (FDR < 0.20) and RNA-seq differential expression (FDR < 0.05).

### Analysis of TCGA-UCEC data

*ARID1A* alteration incidence analysis was calculated using the TCGA Pan-Can UCEC^22^ cohort (*N* = 509) retrieved from cBioPortal^85^. All molecular data for subsequent analyses was pulled from the 28th January, 2016 release of Broad GDAC Firehose (10.7908/C11G0KM9). For molecular comparisons, patients were considered ARID1A^mut^ if they had somatic alterations (excluding missense and synonymous mutations) and ARID1A^wt^ if no alterations were detected at the ARID1A locus. RNASeqV2 RSEM^86^ normalized gene counts were quantile normalized prior to filtering low-count genes (one count per sample on average) and fitting linear models via *limma*^87^ for differential expression analysis in subsets of patients. Moderated statistics were calculated by empirical Bayes moderation via *limma::eBayes* with arguments ‘trend = TRUE’ and ‘robust = TRUE’, and probabilities were adjusted for multiple testing by FDR. Additional metrics for clinical staging and tumor invasion were acquired from the GDC^88^ TCGA-UCEC dataset (*N* = 605) in UCSC Xena^28^. Broad *GSEA*^89^ for mSigDb v6.2 Hallmark pathways^90^ was performed on ortholog-converted *DESeq2* normalized counts from generated mouse data and RNASeqV2 RSEM normalized counts from TCGA-UCEC data. Broad *ssGSEA*^91^ was also performed on RNASeqV2 RSEM normalized counts from TCGA-UCEC data. Orthologs of the mouse gene signature established herein were used to define UCEC endometrioid patients in ssGSEA-enriched or unenriched quartiles, which reflect mouse model transcriptome.

### Bioinformatics and statistics

The 77 gene Pan-Cancer EMT signature was extracted from Supplementary Table S2 of Mak et al.^37^. Various *ClusterProfiler*^92^ functions were used to calculate and visualize pathway enrichment from a list of gene symbols or Entrez^93^ IDs with respective gene universes. *biomaRt*^94,95^ was used for all gene nomenclature and ortholog conversions. *ggplot2*^96^ was used for various plotting applications*. ComplexHeatmap*^97^ was used for hierarchical clustering by Euclidean distance and visualization. *eulerr* was used to produce proportional Euler diagrams^98^. The cumulative hypergeometric distribution was used for enrichment tests performed throughout this manuscript. The statistical computing language R was used for many applications throughout this manuscript^99^. *HOMER* was used to compute integer read counts at loci of interest for tag density heatmaps and scatter plots. *TxDb.Hsapiens.UCSC.hg38.knownGene* was used to generate promoter regions for all standard hg38 genes^100^.

### Transfection of 12Z cells with siRNA and plasmid DNA

12Z cells were seeded at a density of 40,000 cells/mL in DMEM/F12 media supplemented with 10% FBS and 1% l-glutamine. The following day, cells were transfected with 50 pmol/mL of siRNA (Dharmacon, ON-TARGETplus Non-targeting Pool and human ARID1A #8289 SMARTpool) using the RNAiMax (ThermoFisher) lipofectamine reagent according to the manufacturer’s instructions at a ratio of 1:1 volume:volume in OptiMEM (Gibco). After 24 h, the media was replaced. ATAC samples were collected after 48 h. For plasmid co-transfection experiments, 24 h after siRNA transfection, cells were transfected with 500 ng pBabe vector containing PIK3CA^H1047R^ (pPIK3CA^H1047R^) or pBabe empty vector using the FuGene HD transfection reagent (Promega) according to the manufacturers’ instructions at a ratio of 2:1 volume:mass, and media was replaced after 4 h. The pPIK3CA^H1047R^ was a gift from Jean Zhao (Addgene plasmid 12524)^101^. The following day, media was replaced with DMEM/F12 media supplemented with 0.5% FBS, 1% P/S, and 1% l-glutamine. Cells were collected 72 h post siRNA transfection using the Quick-RNA Miniprep Kit (Zymo Research) for RNA or RIPA buffer (Cell Signaling) for protein.

### Generation of lentiviral shRNA particles

Lentiviral particles expressing shRNAs were produced in 293T cells according to the manufacturers’ instructions. Briefly, Lenti-X^TM^ 293T cells were transfected with lentiviral packaging mix (Sigma) and MISSION pKLO.1 plasmid containing non-targeting shRNA (shNONtg) or pooled ARID1A shRNAs (shARID1A) (Sigma) using polyethylenimine (PEI) in DMEM + 4.5 g/L d-Glucose, 110 mg/L Sodium Pyruvate, 10% FBS, 1% l-glutamine. After 4 h, media was replaced with DMEM/F12, 10% FBS, 1% L-glutamine, 1% P/S. Viral particles were collected after 48 and 96 h, and viral titers were calculated using the qPCR Lentiviral Titration Kit (ABM).

### Migration assay

12Z cells were seeded into 35 mm dishes containing four-well culture inserts at a density of 4000 cells per well. After 24 h, cells were transfected with 125 ng pBabe vector or pPIK3CA^H1047R^ using the FuGene HD as described above. After 4 h, cells were treated with lentiviral particles expressing shNONtg or shARID1A at a multiplicity of infection of 100. After 24 h, the media was replaced. At 48 h post transfection, media was replaced with serum-free DMEM/F12 containing 1% l-glutamine and 1% P/S. After 16 h of serum deprivation, culture inserts were removed and serum-free media was added. At 0 and 24 h, images were taken using a Nikon Eclipse T*i* microscope. Distances between migration fronts were measured using NIS Elements Advanced Research software at 16 different points 100 μm apart. Migration distance was calculated by subtracting the average distance across migration fronts at 24 h from the average distance at 0 h. Cells counts were conducted within a 1500 μm by 700 μm window surrounding the migration area.

### Invasion assay

12Z cells were seeded in six-well dishes at a density of 50,000 cells per well. After 24 h, cells were transfected with pPIK3CA or empty vector as described above. After 4 h, cells were treated with lentiviral particles expressing shNONtg or shARID1A at a multiplicity of infection of 100. Media was replaced after 24 h. At 48 h post transfection, cells were trypsinized, and 100 μL of cell mixture containing 30,000 cells and 0.3 mg/mL Matrigel was seeded into transwell plates (8 μm pore polycarbonate membrane, Corning) pre-coated with 100 μL of 0.3 mg/mL Matrigel. After 1 h, serum-free DMEM/F12 1% P/S, 1% l-glutamine media was added to the top chamber and DMEM/F12, 5% FBS, 1% P/S, 1% l-glutamine was added to the bottom chamber. After 16 h, transwell units were transferred to plates containing 4 μg/mL calcein AM in DMEM/F12. After 1 h, media was aspirated from the top chamber and unmigrated cells were removed with a cotton swab. Images were collected using a Nikon Eclipse T*i* microscope in five non-overlapping fields per well. ImageJ software (National Institutes of Health) was used to quantify cells based on size and intensity.

### Western blotting

Protein lysates were quantified using the Micro BCA Protein Assay Kit (ThermoFisher) and a FlexSystem3 plate reader. Protein lysates were run on a 4–15% gradient sodium dodecyl sulfate polyacrylamide gel electrophoresis (SDS-PAGE) gel (BioRad) and transferred to PVDF membrane using the TransBlot Turbo system (BioRad). Primary antibodies were used at the following dilutions: 1:1000 ARID1A (D2A8U) (12354, Cell Signaling); 1:1000 Akt (4691, Cell Signaling); 1:1000 β-Actin (8457, Cell Signaling); E-Cadherin (3195, Cell Signaling); 1:2000 Phospho-Akt (Ser473) (4060, Cell Signaling); 1:1000 Slug (9585, Cell Signaling); 1:1000 Snail (3879, Cell Signaling); 1:1000 Twist1 (T6451, Sigma); 1:100 ARID1B (sc-32762, Santa Cruz); 1:1000 Brg1 (ab110641, Abcam); 1:1000 BRM (11966, Cell Signaling); 1:100 ARID1A (PSG3) (sc-32761, Santa Cruz). Horseradish peroxidase (HRP) conjugated secondary antibodies (Cell Signaling) were used at a dilution of 1:2000. Clarity Western ECL Substrate (BioRad) was used for protein band visualization, and western blot exposures were captured using the ChemiDoc XRS + imaging system (BioRad). Uncropped western blot images can be found in Supplementary Fig. 7.

### Chromatin immunoprecipitation

Wild-type 12Z cells were treated with 1% formaldehyde in DMEM/F12 media for 10 min at room temperature. Formaldehyde was quenched by the addition of 0.125 M Glycine and incubation for 5 min at room temperature, followed by wash with PBS. In all, 1 × 10^7^ crosslinked cells were used per IP. Chromatin from crosslinked cells was fractionated by digestion with micrococcal nuclease using the SimpleChIP Enzymatic Chromatin IP Kit (Cell Signaling) as per the manufacturers’ instructions, followed by 30 s of sonication. IPs were performed using the SimpleChIP Enzymatic Chromatin IP Kit per the manufacturers’ instructions with 1:100 anti-ARID1A (D2A8U) (12354, Cell Signaling). Crosslinks were reversed with 0.4 mg/mL Proteinase K (ThermoFisher) and 0.2 M NaCl at 65 °C for 2 h. DNA was purified using the ChIP DNA Clean & Concentrator Kit (Zymo).

### Chromatin immunoprecipitation sequencing (ChIP-seq)

Libraries for input and IP samples were prepared by the Van Andel Genomics Core from 10 ng of input material and IP material using the KAPA Hyper Prep Kit (v5.16) (Kapa Biosystems, Wilmington, MA USA). Prior to PCR amplification, end repaired and A-tailed DNA fragments were ligated to Bioo Scientific NEXTflex Adapters (Bioo Scientific, Austin, TX, USA). Quality and quantity of the finished libraries were assessed using a combination of Agilent DNA High Sensitivity chip (Agilent Technologies, Inc.), QuantiFluor dsDNA System (Promega Corp., Madison, WI, USA), and Kapa Illumina Library Quantification qPCR assays (Kapa Biosystems). Individually indexed libraries were pooled and 75 bp, single-end sequencing was performed on an Illumina NextSeq 500 sequencer using 75 cycle HO sequencing kits (v2) (Illumina Inc., San Diego, CA, USA), with all libraries run across two flow cells to return a minimum read depth of 80 M reads per input library and 40 M read per IP library. Base calling was done by Illumina NextSeq Control Software (NCS) v2.0 and output of NCS was demultiplexed and converted to FastQ format with Illumina Bcl2fastq v1.9.0.

### ChIP-seq analysis

Technical replicate libraries were combined across flow cells and trimmed with *cutadapt* and *Trim Galore!* followed by quality control analysis via *FastQC*. Trimmed reads were aligned to GRCh38.p12 reference genome via *Bowtie2*^74^ with flag ‘–very-sensitive’. Reads were then sorted and indexed with *samtools*^76^. PCR duplicates were removed with *Picard MarkDuplicates* (http://broadinstitute.github.io/picard/), and again sorted and indexed. *MACS2*^80^ was used to call broad peaks with FDR < 0.05 threshold on each ChIP replicate against the input control, and the resulting peaks were repeat-masked by blacklist filtering^81^. A naive overlap peak set, as defined by ENCODE, was constructed by combining replicates and calling broad peaks on pooled BAM files followed by *intersectBed* to select for peaks of at least 50% overlap with each biological replicate. Naive overlapping ChIP peaks were annotated by *HOMER*, and de novo motif enrichment and genome ontology were performed on genome-wide and promoter (within 3 kb of a TSS) peak sets. Overlapping genes between ChIP/ATAC and ChIP/ATAC/RNA were selected by the presence of a significant ChIP peak and differentially accessible promoter ATAC peak (FDR < 0.05) located in the same promoter region (within 3 kb of TSS).

### Co-immunoprecipitation (co-IP)

Small-scale nuclear extracts and co-IPs from wild-type 12Z cells were performed^20^. Briefly, Protein A or Protein G Dynabeads (Invitrogen) were conjugated with anti-ARID1A (D2A8U) (12354, Cell Signaling) anti-ARID1A (PSG3) (sc-32761, Santa Cruz), or anti-ARID1B (E9J4T) (92964, Cell Signaling) in PBS + 0.5% BSA overnight at 4 C. Four-hundred micrograms of nuclear lysate was added to a final volume of 1 mL IP buffer (20 mM HEPES [pH 7.9], 250 mM KCl, 10% glycerol, 0.2 mM EDTA, 0.1% Tween-20, 0.5 mM DTT, 0.5 mM PMSF), clarified by high-speed centrifugation and added to antibody-conjugated beads (D2A8U, 1:200; PSG3, 1:40; E9J4T, 1:200) and incubated overnight at 4 °C. IP samples were washed in a series of IP buffers with varying salt concentrations as follows: 150 mM KCl, 300 mM KCl, 500 mM KCl, 300 mM KCl, 100 mM KCl. IP samples were washed a final time in 60 mM KCl IP buffer in the absence of EDTA or Tween-20. Proteins were eluted twice with 100 mM glycine pH 2.5 on ice and neutralized by the addition of 1:10 (v:v) of 1 M Tris-HCl pH 8.0.

### Co-IP followed by mass spectrometry

Nuclear lysates from wild-type 12Z cells were prepared as described in the previous section. Protein A Dynabeads (Invitrogen) were conjugated with 8.3 μg anti-ARID1A (D2A8U) (12354, Cell Signaling) or IgG (2729, Cell Signaling) in PBS + 0.5% BSA + 0.01% Tween-20 overnight at 4 °C. Antibody-bead conjugates were crosslinked in BS^3^ (ThermoFisher) as described by the manufacturer protocol, and excess unlinked antibody was removed by one wash of 0.11 M glycine followed by quenching with Tris-HCl. 4.3 mg of nuclear lysate was added to a final volume of 14 mL IP buffer (20 mM HEPES [pH 7.9], 150 mM KCl, 10% glycerol, 0.2 mM EDTA, 0.1% Tween-20, 0.5 mM DTT, 0.5 mM PMSF) and clarified by high-speed centrifugation. Diluted nuclear lysate was added to antibody-crosslinked beads and incubated overnight at 4 °C. IP samples were washed in an IP buffer series with varying salt concentrations as follows: twice with 150 mM KCl, three times with 300 mM KCl, twice with 100 mM KCl. IP samples were washed a final time in 60 mM KCl IP buffer in the absence of EDTA or Tween-20. Proteins were eluted in 2x Laemmli + 100 µM DTT at 70 °C for 10 min. Eluates were processed for short-gel SDS-PAGE and mass spectrometry by the University of Massachusetts Mass Spectrometry core.

### Mass spectrometry analysis

All MS/MS samples were analyzed using Mascot (version 2.1.1.21, Matrix Science, London, UK). Mascot was set-up to search UniProtKB Swiss-Prot (Human) assuming the digestion enzyme as strict trypsin. Mascot was searched with a fragment ion mass tolerance of 0.050 Da and a parent ion tolerance of 10.0 PPM. Carbamidomethyl of cysteine was specified in Mascot as a fixed modification. Gln- > pyro-Glu of glutamine and the N-terminus, oxidation of methionine and acetyl of the N-terminus were specified in Mascot as variable modifications. Scaffold (version 4.8.8, Proteome Software Inc., Portland, OR) was used to validate MS/MS-based peptide and protein identifications. Peptide identifications were accepted if they could be established at >85.0% probability by the Peptide Prophet algorithm^102^ with Scaffold delta-mass correction. Protein identifications were accepted if they could be established at greater than 99.0% probability and contained at least two identified peptides. Protein probabilities were assigned by the Protein Prophet algorithm^103^. Proteins that contained similar peptides and could not be differentiated based on MS/MS analysis alone were grouped to satisfy the principles of parsimony. Proteins sharing significant peptide evidence were grouped into clusters.

### Reporting summary

Further information on research design is available in the Nature Research Reporting Summary linked to this article.

## Supplementary information

Supplementary Information

Reporting Summary

## Data Availability

The RNA-seq, ChIP-seq and ATAC-seq data have been deposited in the GEO database under the accession code GSE121198. All the other data supporting the findings of this study are available within the article and its supplementary information files and from the corresponding author upon reasonable request. Uncropped western blot images can be found in Supplementary Fig. 7. A reporting summary for this article is available as a Supplementary Information file.
