# Supplementary material for: ARID1A and PI3-kinase pathway mutations in the endometrium drive epithelial transdifferentiation and collective invasion
Source: Nat Commun. 2019 Aug 7;10:3554. doi: 10.1038/s41467-019-11403-6 (PMC6686004; doi:10.1038/s41467-019-11403-6)
Supplement: Supplementary file 1 — Supplementary Information [file 41467_2019_11403_MOESM1_ESM.pdf]

ARID1A and PI3-Kinase pathway mutations in the endometrium drive epithelial transdifferentiation and collective invasion

Wilson & Reske et al.

Supplementary Information

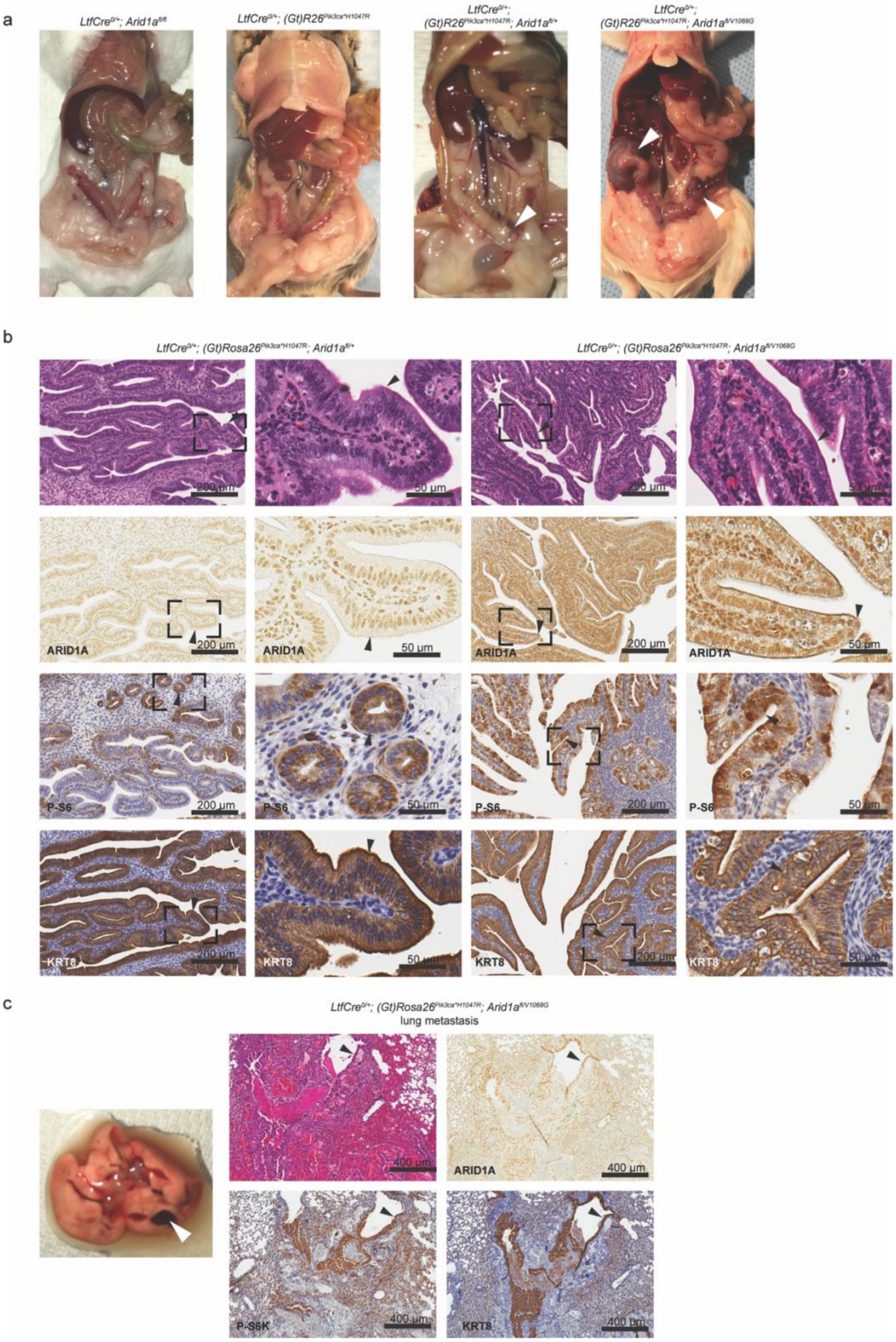

Supplementary Fig. 1 | Phenotypic analysis of *LtfCre$^{0/+}$; (Gt)R26Pik3ca^{*H1047R}; Arid1a$^{fl/+}$*, *LtfCre$^{0/+}$; (Gt)R26Pik3ca^{*H1047R}; Arid1a$^{fl/V1068G}$* and lung metastasis. a, Representative gross images of mice at time of sacrifice due to vaginal bleeding. White arrows indicate tumors. Size

of uterine tumor varies dramatically within genotype at time of sacrifice. **b**, H&E staining and IHC for ARID1A, P-S6 and KRT8 ($n \geq 2$) of the endometrium at 5X (scale bar = 200 μm) and 20X (scale bar = 50 μm) magnification, with 20X magnifications representing portion panel to the right surrounded by black box. ARID1A expression is retained in the endometrial epithelium of *LtfCre*$^{0/+}$*; (Gt)R26Pik3ca*^{*H1047R}*; Arid1a*$^{fl/+}$ and *LtfCre*$^{0/+}$*; (Gt)R26Pik3ca*^{*H1047R}*; Arid1a*$^{fl/V1068G}$. P-S6 is shown as marker of AKT pathway activation, and KRT8 as a marker of endometrial epithelium. Arrows indicate endometrial epithelium. **c**, Gross image, histology and IHC of *LtfCre*$^{0/+}$*; (Gt)R26Pik3ca*^{*H1047R}*; Arid1a*$^{fl/V1068G}$ lung metastasis observed in one mouse. White arrow indicates lung metastasis. Black arrows indicate mutant epithelium (scale bar = 400 μm).

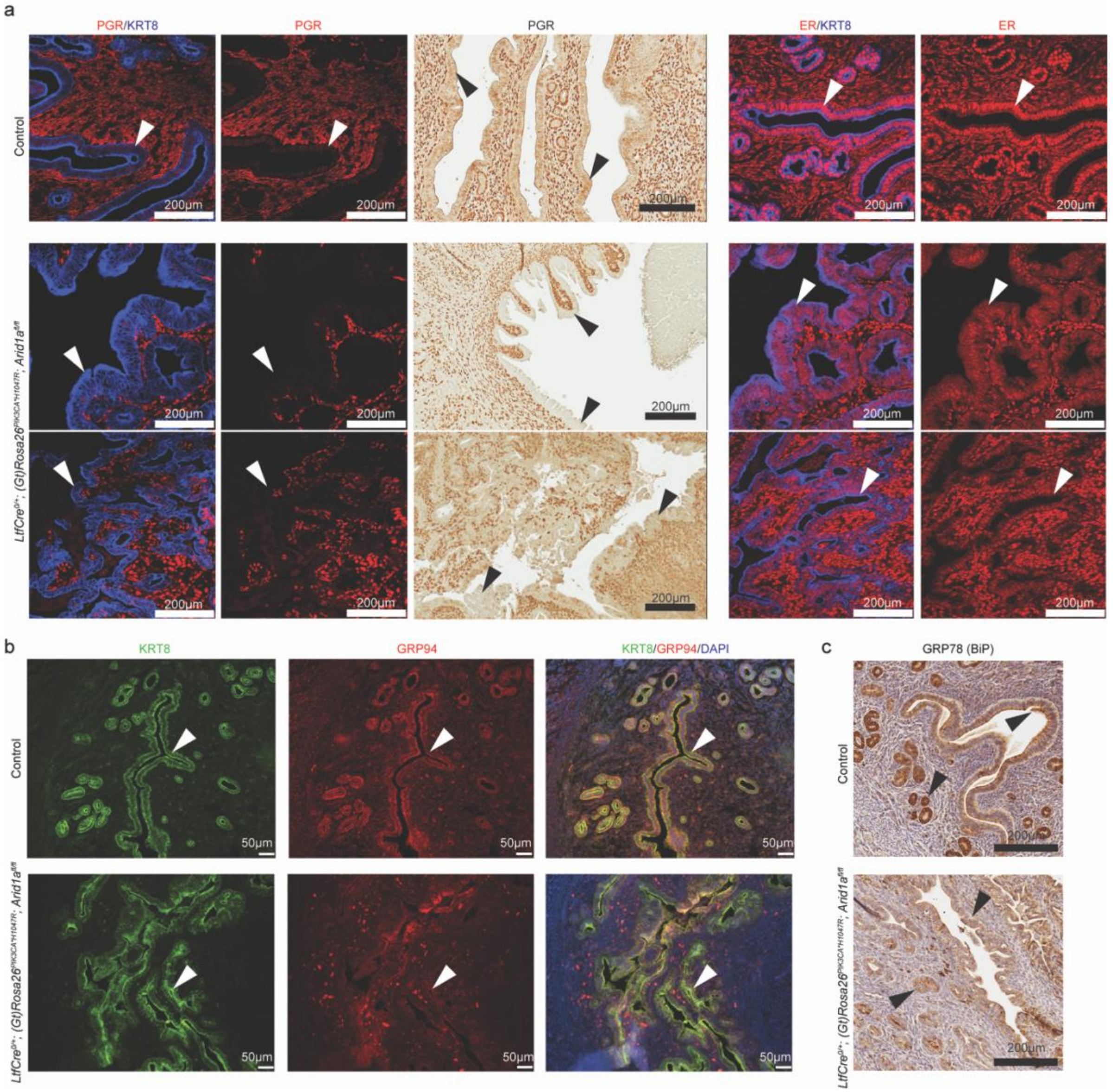

Supplementary Fig. 2 | *LtfCre*$^{0/+}$*; (Gt)R26Pik3ca*^{*H1047R}*; Arid1a*$^{fl/fl}$ endometrium shows downregulation of steroid hormone receptors and unfolded protein response proteins. a, IF and IHC images detailing changes in hormone receptor expression in *LtfCre*$^{0/+}$*; (Gt)R26Pik3ca*^{*H1047R}*; Arid1a*$^{fl/fl}$ (N ≥ 2). KRT8 identifies endometrial epithelium. Confocal IF images represent maximum intensity projections. Arrows indicate endometrial epithelium (scale bar = 200 μm). **b**, IF images detailing changes in unfolded protein response protein GRP94 in *LtfCre*$^{0/+}$*; (Gt)R26Pik3ca*^{*H1047R}*; Arid1a*$^{fl/fl}$ endometrium, counterstained with KRT8 and DAPI (n ≥ 2). Arrows indicate endometrial epithelium (scale bar = 50 μm). **c**, IHC images detailing changes in unfolded protein response protein GRP78 in *LtfCre*$^{0/+}$*; (Gt)R26Pik3ca*^{*H1047R}*; Arid1a*$^{fl/fl}$ endometrium (n ≥ 2). Arrows indicate endometrial epithelium (scale bar = 200 μm).

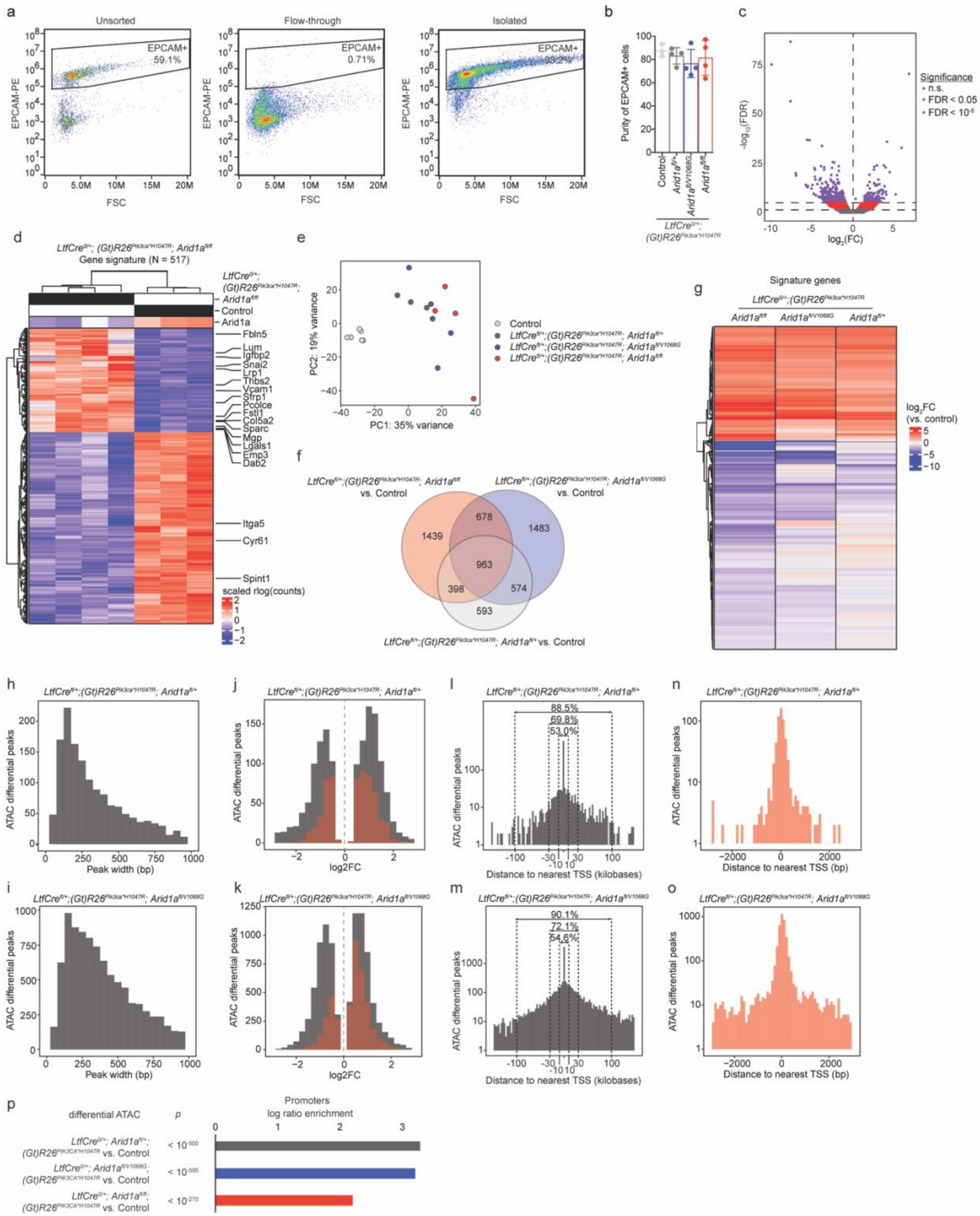

Supplementary Fig. 3 | Allelic series of ARID1A loss with *(Gt)R26Pik3ca*^{*H1047R} in endometrial epithelium displays concordant gene expression and differential chromatin accessibility. a, Flow-cytometry analysis of EPCAM purity (based on PE labeling) before and

after sorting. **b**, Purity of EPCAM-isolated cell populations by genotype (N = 3 for control, N = 4 for other genotypes). No significant difference in purity was observed between genotypes (two-sided unpaired *t*-test, plot represents mean ± s.d). **c**, Volcano plot of RNA-seq differential gene dataset from EPCAM-isolated cells. Purple dots represent genes with significance FDR < 10^{-5} and represent the 517 gene signature for further analysis (genes with $|\log_2 FC| < 1$ subsequently filtered). **d**, Clustering of 517 differentially-expressed signature genes. EMT genes from Hallmark pathway and Mak & Tong pan-cancer gene signature are identified. **e,** Principal component analysis of gene expression data from *LtfCre*$^{0/+}$; *(Gt)R26Pik3ca*^{*H1047R}; *Arid1a*$^{fl/fl}$, *LtfCre*$^{0/+}$; *(Gt)R26Pik3ca*^{*H1047R}; *Arid1a*$^{fl/+}$ and *LtfCre*$^{0/+}$; *(Gt)R26Pik3ca*^{*H1047R}; *Arid1a*$^{fl/V1068G}$ and control EPCAM-sorted endometrial epithelial cells. **f**, Proportional Euler diagram displaying differentially-expressed genes from *LtfCre*$^{0/+}$; *(Gt)R26Pik3ca*^{*H1047R}; *Arid1a*$^{fl/fl}$, *LtfCre*$^{0/+}$; *(Gt)R26Pik3ca*^{*H1047R}; *Arid1a*$^{fl/+}$ and *LtfCre*$^{0/+}$; *(Gt)R26Pik3ca*^{*H1047R}; *Arid1a*$^{fl/V1068G}$. Significant overlap between differentially-expressed gene comparisons (FDR < 0.05) for individual experimental genotypes vs. control was observed. Only significant genes with concordant directionality of change between comparisons were considered intersecting. **g**, Hierarchical clustering of $\log_2$FC (vs. control) values for *LtfCre*$^{0/+}$; *(Gt)R26Pik3ca*^{*H1047R}; *Arid1a*$^{fl/fl}$ signature genes (Fig. 2g) within each genotypic comparison. **h-i**, Peak width distribution of differentially-accessible peaks (FDR < 0.20) for *LtfCre*$^{0/+}$; *(Gt)R26Pik3ca*^{*H1047R}; *Arid1a*$^{fl/+}$ (h) and *LtfCre*$^{0/+}$; *(Gt)R26Pik3ca*^{*H1047R}; *Arid1a*$^{fl/V1068G}$ (i). **j-k**, Magnitude distribution of differentially-accessible peaks separated by total peaks and promoter peaks for *LtfCre*$^{0/+}$; *(Gt)R26Pik3ca*^{*H1047R}; *Arid1a*$^{fl/+}$ (j) and *LtfCre*$^{0/+}$; *(Gt)R26Pik3ca*^{*H1047R}; *Arid1a*$^{fl/V1068G}$ (k). **l-m**, Histogram of all differential ATAC peaks for *LtfCre*$^{0/+}$; *(Gt)R26Pik3ca*^{*H1047R}; *Arid1a*$^{fl/+}$ (l) and *LtfCre*$^{0/+}$; *(Gt)R26Pik3ca*^{*H1047R}; *Arid1a*$^{fl/V1068G}$ (m) depicting distance to nearest TSS. Percent of peaks found within +/- 10, 30, or 100 kb of the TSS are shown. **n-o**, Histogram of differential ATAC promoter peaks for *LtfCre*$^{0/+}$; *(Gt)R26Pik3ca*^{*H1047R}; *Arid1a*$^{fl/+}$ (n) and *LtfCre*$^{0/+}$; *(Gt)R26Pik3ca*^{*H1047R}; *Arid1a*$^{fl/V1068G}$ (o) depicting distance to nearest TSS. **p,** Significant enrichment for promoters among differentially-accessible peaks in *LtfCre*$^{0/+}$; *(Gt)R26Pik3ca*^{*H1047R}; *Arid1a*$^{fl/fl}$, *LtfCre*$^{0/+}$; *(Gt)R26Pik3ca*^{*H1047R}; *Arid1a*$^{fl/+}$ and *LtfCre*$^{0/+}$; *(Gt)R26Pik3ca*^{*H1047R}; *Arid1a*$^{fl/V1068G}$. Enrichment ratio is calculated by promoter bp in ATAC peak set compared to background genome.

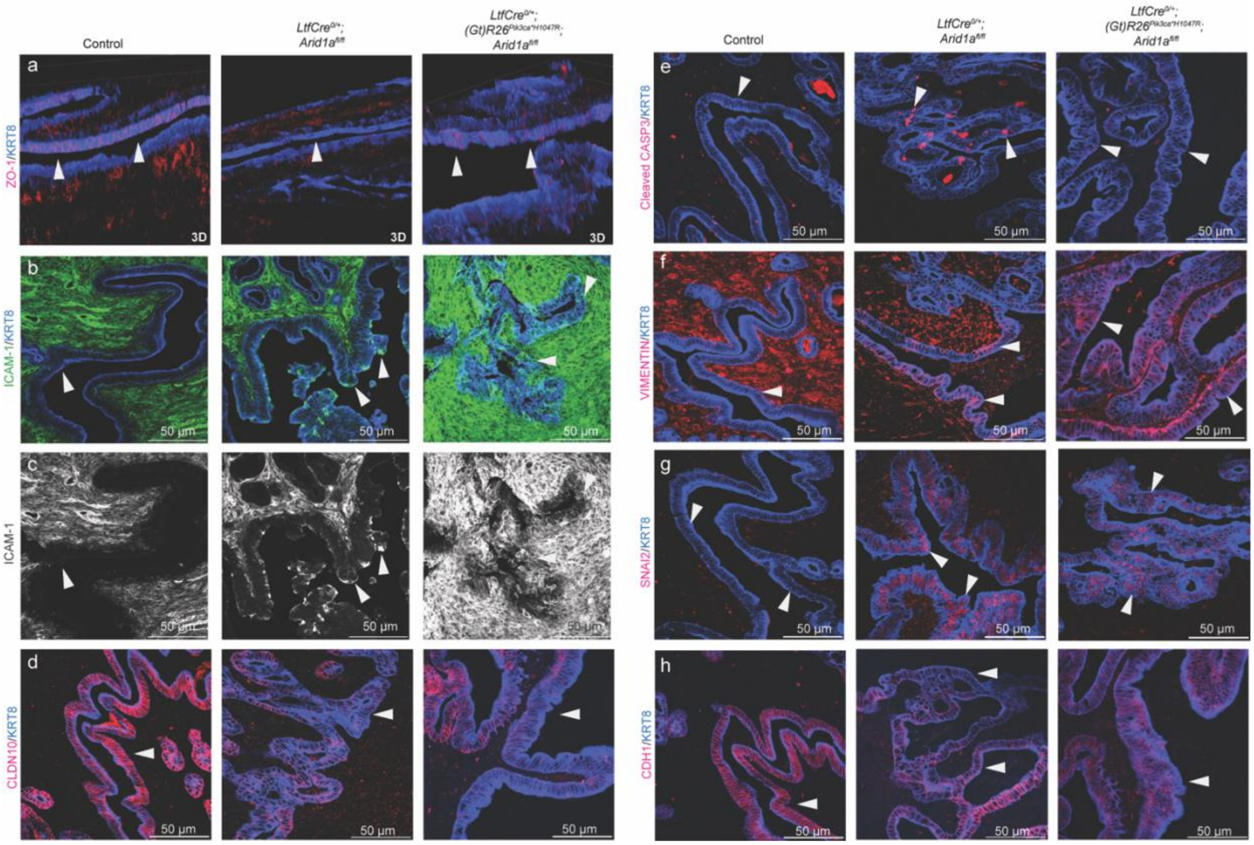

Supplementary Fig. 4 | ARID1A loss induces EMT in mouse endometrium. a-h, Images of maximum intensity confocal projections of control, *LtfCre*$^{0/+}$*; Arid1a*$^{fl/fl}$ and *LtfCre*$^{0/+}$*; (Gt)R26Pik3ca*^{*H1047R}*; Arid1a*$^{fl/fl}$ endometrium sections stained with ZO-1 (a), ICAM-1 (b-c) CLDN10 (d), Cleaved CASP3 (e), VIM (f), SNAI2 (g), CDH1 (h) ($n \geq 2$). To label endometrial epithelium, the slides were counter-stained with KRT8. White arrows indicate endometrial epithelium (scale bar = 50 µm).

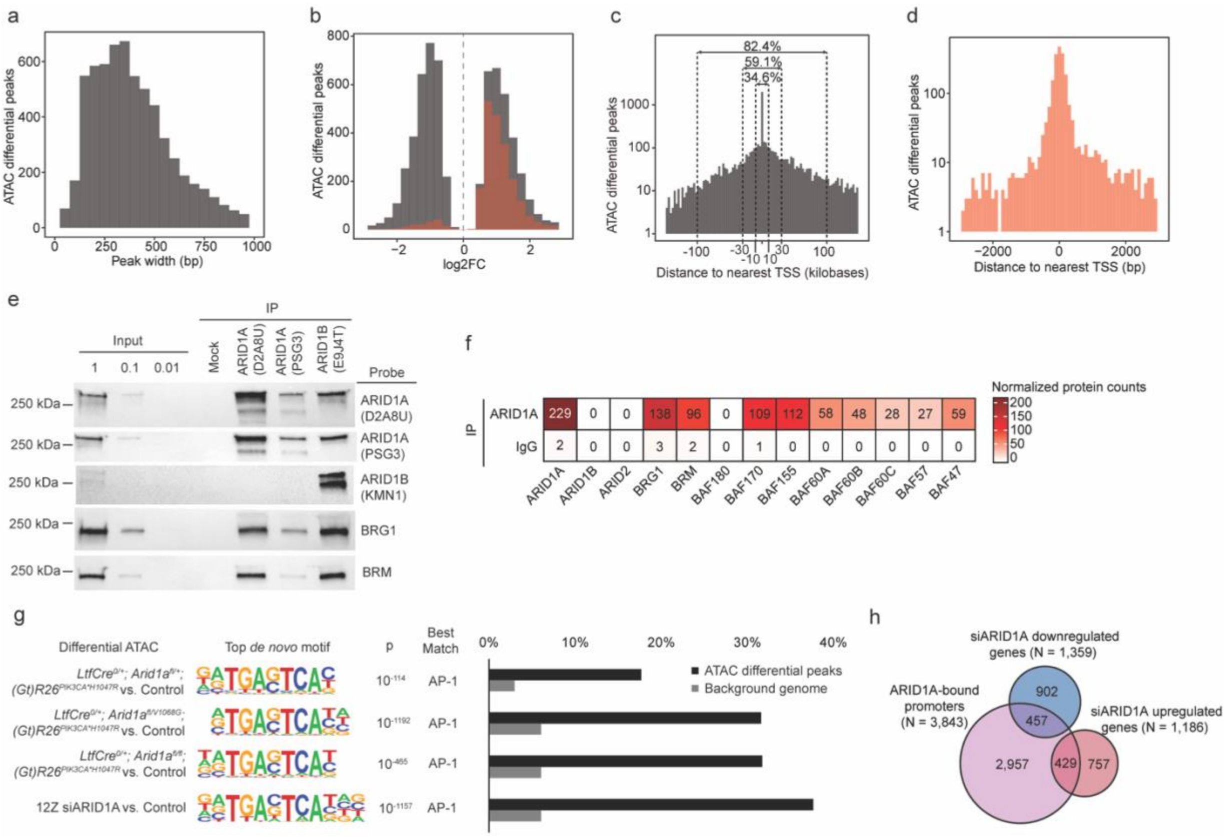

Supplementary Fig. 5 | Characterization of 12Z ATAC-seq and ARID1A ChIP antibody. a, Peak width distribution of differentially-accessible peaks for 12Z siARID1A vs. control (FDR < 0.05). **b**, Magnitude distribution of differentially-accessible peaks separated by genome-wide peaks and promoter peaks for 12Z siARID1A. **c**, Histogram of all differential ATAC peaks for 12Z siARID1A depicting distance to nearest TSS. Percent of peaks found within +/- 10, 30, or 100 kb of the TSS are shown. **d**, Histogram of differential ATAC promoter peaks for 12Z siARID1A depicting distance to nearest TSS. **e,** Immunoprecipitation of ARID1A and SWI/SNF subunits using anti-ARID1A (D2A8U) (12354, Cell Signaling), anti-ARID1A (PSG3) (sc-32761, Santa Cruz) or anti-ARID1B (E9J4T) (92964, Cell Signaling) antibodies. **f,** Mass spectrometry analysis of SWI/SNF subunits found in anti-ARID1A (D2A8U) immunoprecipitation sample. **g,** HOMER motif enrichment of differentially-accessible peaks found in *LtfCre$^{0/+}$; (Gt)R26Pik3ca^{*H1047R}; Arid1a$^{fl/fl}$*, *LtfCre$^{0/+}$; (Gt)R26Pik3ca^{*H1047R}; Arid1a$^{fl/+}$*, *LtfCre$^{0/+}$; (Gt)R26Pik3ca^{*H1047R}*; *Arid1a$^{fl/V1068G}$* and 12Z siARID1A samples. **h**, Proportional Euler diagram of overlap between ARID1A promoter binding, decreasing and increasing gene expression.

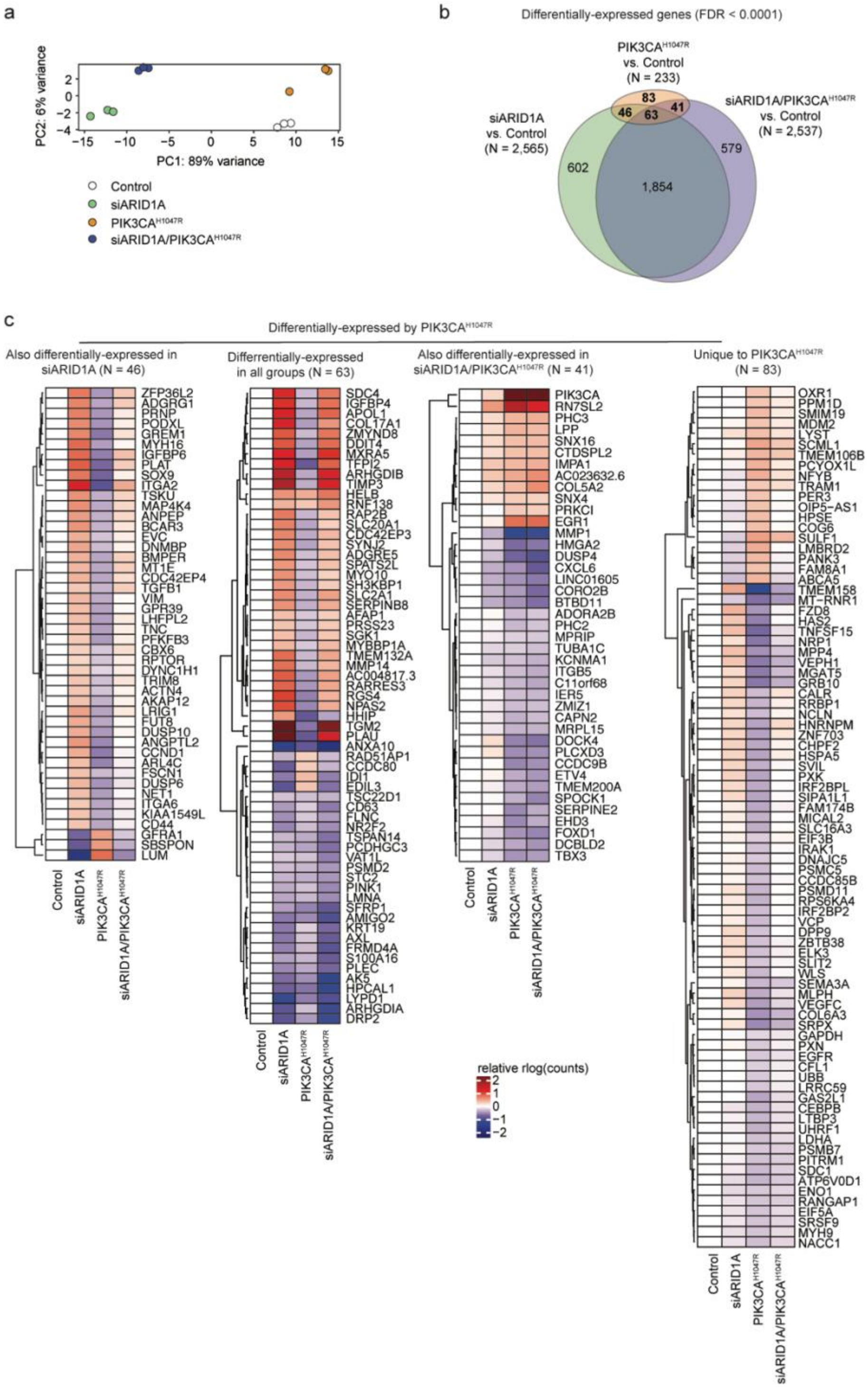

Supplementary Fig. 6 | Differential gene expression as a result of PIK3CA^{H1047R} overexpression. a, Principal component analysis of gene expression data from 12Z control, siARID1A, PIK3CA^{H1047R}, and siARID1A/PIK3CA^{H1047R} samples. **b,** Proportional Euler diagram displaying differentially-expressed genes from siARID1A, PIK3CA^{H1047R}, and siARID1A/PIK3CA^{H1047R}. **c,** Hierarchical clustering of relative gene expression within each overlapping group (Supplemental Fig. 5a) segregated by differential expression in siARID1A, PIK3CA^{H1047R}, and siARID1A/PIK3CA^{H1047R}.

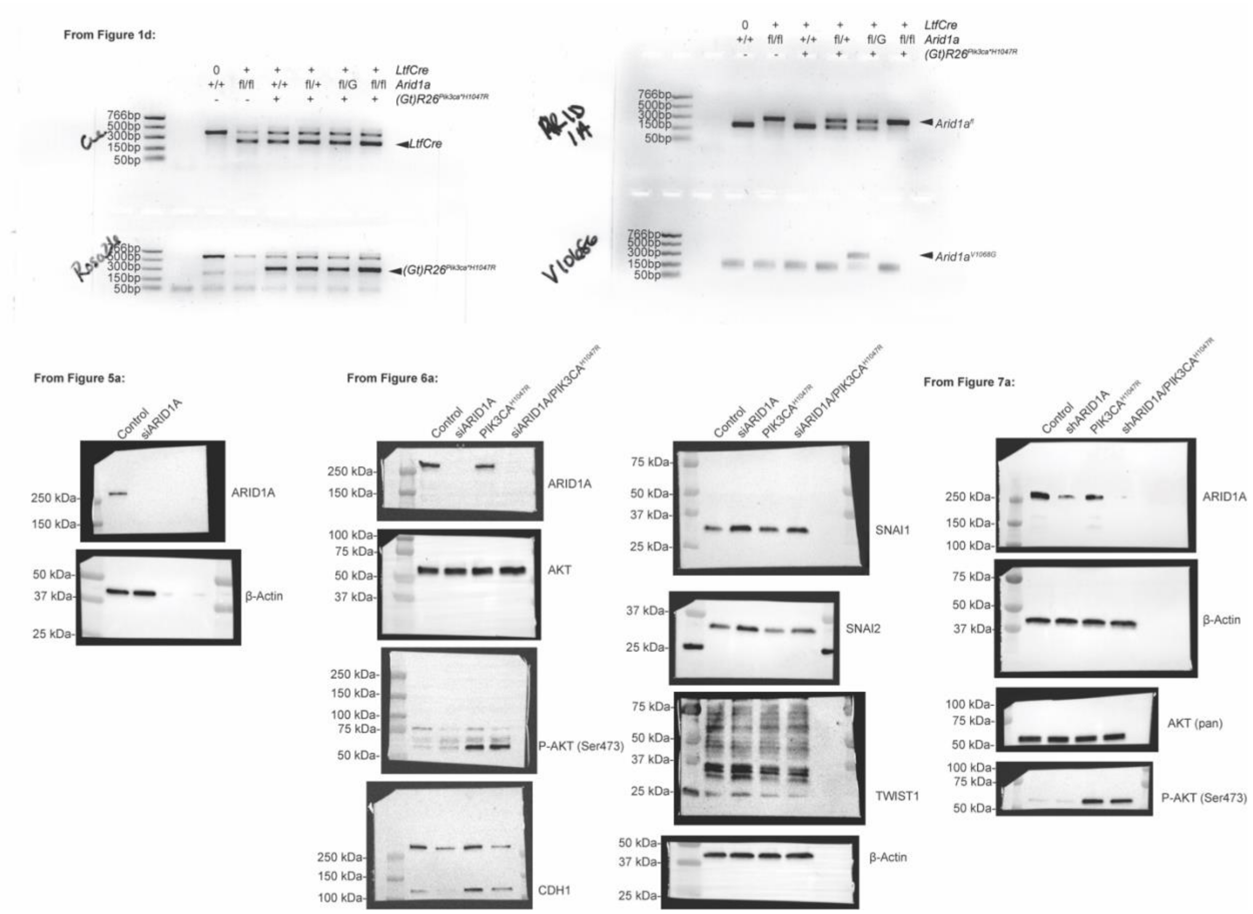

Supplementary Fig. 7 | Uncropped gels and blots from figures in main text.

Supplementary Table 1 | List of primers used for PCR

Mouse allele	Forward primer	Reverse primer
LtfCre	AACTAGCACACCTGGTTGAGG	CAGGTTTTGGTGCACAGTCA
(Gt)R26LacZ	GGACTGGGTGGATCAGTCGCTGA	GTGGAGCGACATCCAGAGGCACT
(Gt)R26Pik3ca^{*H1047R}	AAAGTCGCTCTGAGTTGTTAT	GCGAAGAGTTTGTCCTCAACC
Arid1a^{fl}	CTAGGTGGAAGGTAGCTGACTGA	TACACGGAGTCAGGCTGAGC
Arid1a^{V1068G}	GCCGTTTGAATTTGTCCTGT	TCTTGGGTTGGGACTTCTTG

Human gene	Forward primer	Reverse primer
hANTXR1	GCCTCCACAAAATTGCATCAG	GGAAAGTGTAAACCGAGCATG
hARID1A	AGATGGGACACCCAAGACAG	CTTCCTCTCAGGCTCACCAC
hCCND1	CATCTACACCGACAACTCCATC	TCTGGCATTTTGGAGAGGAAG
hCDH1	CTGCCAATCCCGATGAAATTG	TCCTTCATAGTCAAACACGAGC
hCDH2	CCCAAGACAAAGAGACCCAG	GCCACTGTGCTTACTGAATTG
hCOL1A2	GATTGAGACCCTTCTTACTCCTGAA	GGGTGGCTGAGTCTCAAGTCA
hCOL6A1	TCAAGTCCTTCACCAAGCG	ATCTCCACCTCGTCACTGTA
hCOL6A2	GACCAGGACACCATCAACC	TCACCCATGTTGCCCTTG
hFAP	AGCTTCCTCGTCCAATTCAG	TGGATCTCCTGGTCTTTGTTTC
hINHBA	ACGGGTATGTGGAGATAGAGG	TGGAAATCTCGAAGTGCAGC
hLOX	ACATTCGCTACACAGGACATC	TTCCCACTTCAGAACACCAG
hLOXL2	AGGTTGCAGAATCCGATTACTC	TGTTTAAGAGCCCGCTGAAG
hOLFML2B	GCAACACCCTGGTAGAGTTC	TACGGGAGCTTGTAGGAATTG
hRPL17	GACCTTGTGTCCAGCCCCAT	ACGAAAAGCCACGAAGTATCTG
hSNAI1	ACAAGCACCAAGAGTCCG	ATGGCAGTGAGAAGGATGTG
hSNAI2	AGCATTTCAACGCCTCCA	GGATCTCTGGTTGTGGTATGAC
hTWIST1	AGCAAGATTCAGACCCTCAAG	ACTGTCCATTTTCTCCTTCTCTG
hVCAM1	TCTACGCTGACAATGAATCCTG	AGGGCCACTCAAATGAATCTC
hVIM	CGTGAATACCAAGACCTGCTC	GGAAAAGTTTGGAAGAGGCAG
hZEB1	AAGTGGCGGTAGATGGTAATGT	AAGGAAGACTGATGGCTGAAAT

Supplementary Table 2 | Key resources table

Reagent or resource	Source	Identifyer
Antibodies		
Rabbit monoclonal anti-ARID1A/BAF250A (D2A8U)	Cell Signaling Technology	Cat# 12354; RRID: AB_2637010
Rabbit monoclonal anti-β-Actin (D6A8)	Cell Signaling Technology	Cat# 8457; RRID: AB_10950489
Rabbit monoclonal anti-Akt (pan) (C67E7)	Cell Signaling Technology	Cat# 4691; RRID: AB_915783
Rabbit monoclonal anti-Phospho-Akt (Ser473) (D9E)	Cell Signaling Technology	Cat# 4060; RRID: AB_2315049
Rabbit monoclonal anti-Phospho-S6 Ribosomal Protein (Ser235/236) (D57.2.2E)	Cell Signaling Technology	Cat# 4858; RRID: AB_2721245
Rabbit monoclonal anti-BiP (C50B12)	Cell Signaling Technology	Cat# 3177; RRID: AB_10828008
Rabbit monoclonal anti-E-Cadherin (24E10)	Cell Signaling Technology	Cat# 3195; RRID: AB_10694492
Rabbit monoclonal anti-Vimentin (D21H3)	Cell Signaling Technology	Cat# 5741; RRID: AB_10695459
Rabbit monoclonal anti-Grp94 (D6X2Q)	Cell Signaling Technology	Cat# 20292; RRID: AB_2722657
Rabbit monoclonal anti-Snail (C15D3)	Cell Signaling Technology	Cat# 3879; RRID: AB_10828214
Rabbit monoclonal anti-Slug (C19G7)	Cell Signaling Technology	Cat# 9585; RRID: AB_10828257
Rabbit polyclonal anti-TWIST1	Cell Signaling Technology	Cat# 46702
Rabbit monoclonal anti-Estrogen Receptor Alpha (E115)	Abcam	Cat# ab32063; RRID: AB_732249
Rabbit monoclonal anti-Progesterone Receptor (SP2)	Sigma-Aldrich	Cat# SAB5500165
Rabbit monoclonal anti-Cleaved Caspase-3 (Asp175) (D3E9)	Cell Signaling Technology	Cat# 9579; RRID: AB_10897512
Rabbit polyclonal anti-Claudin-10	Thermo Fisher Scientific	Cat# 38-8400; RRID: AB_2533386
Rabbit polyclonal anti-ZO-1	Thermo Fisher Scientific	Cat# 61-7300; RRID: AB_2533938
Rat monoclonal anti-TROMA-I	Developmental Studies Hybridoma Bank	Cat# TROMA-I; RRID: AB_531826
Rat monoclonal anti-mouse Ep-CAM	Developmental Studies Hybridoma Bank	Cat# G8.8; RRID: AB_2098655
Mouse monoclonal anti-Actin α-Smooth Muscle Cy3	Sigma	Cat# C6198; AB_476856
Goat polyclonal anti-ICAM-1/CD54	R&D Systems	Cat# AF796-SP; RRID: AB_2248703
Rabbit monoclonal anti-BRG1 (EPNCIR111A)	Abcam	Cat# ab110641; RRID: AB_10861578
Rabbit monoclonal anti-BRM (D9E8B)	Cell Signaling Technology	Cat# 11966
Mouse monoclonal anti-ARID1B (KMN1)	Santa Cruz Biotechnology	Cat# sc-32762; RRID: AB_2060367
Mouse monoclonal anti-ARID1A (PSG3)	Santa Cruz Biotechnology	Cat# sc-32761; RRID: AB_673396

Rabbit monoclonal anti-ARID1B (E9J4T)	Cell Signaling Technology	Cat# 92964;
Goat anti-rabbit IgG, HRP-linked Antibody	Cell Signaling Technology	Cat# 7074; RRID: AB_2099233
Horse anti-mouse IgG, HRP-linked Antibody	Cell Signaling Technology	Cat# 7076; RRID: AB_330924
Donkey anti-rabbit IgG, IRDye 800CW conjugated antibody	LI-COR Biosciences	Cat# 926-32213; RRID: AB_621848
Donkey anti-mouse IgG, IRDye 680LT conjugated	LI-COR Biosciences	Cat# 925-68022
Donkey anti-Rabbit IgG, Biotin-SP-conjugated	Jackson ImmunoResearch Labs	Cat# 711-065-152; RRID: AB_2340593
Donkey anti-Rat IgG, Biotin-SP-conjugated	Jackson ImmunoResearch Labs	Cat# 712-065-153; RRID: AB_2315779
Anti-Rabbit IgG, Alexa Fluor 555-conjugated Antibody	Thermo Fisher Scientific	Cat# A-31572; RRID: AB_162543
Anti-Rabbit IgG, Alexa Fluor 555-conjugated Antibody	Thermo Fisher Scientific	Cat# A-21428; RRID: AB_2535849
Anti-Rat IgG, Alexa Fluor 647-conjugated Antibody	Thermo Fisher Scientific	Cat# A-21247; RRID: AB_141778
Anti-Rat IgG, Alexa Fluor 647-conjugated Antibody	Jackson ImmunoResearch Labs	Cat# 712-605-153; RRID: AB_2340694
Anti-Goat IgG, Alexa Fluor 488-conjugated Antibody	Jackson ImmunoResearch Labs	Cat# 705-545-488; RRID: AB_2336933
Anti-CD326 (EpCAM)-PE, mouse (caa7-9G8)	Miltenyi Biotec	Cat# 130-102-265
Normal Rabbit IgG	Cell Signaling Technology	Cat# 2729; RRID: AB_1031062
Chemicals, Peptides, and Recombinant Proteins		
Normal Goat Serum	Jackson ImmunoResearch Labs	Cat# 005-000-121
Normal Donkey Serum	Jackson ImmunoResearch Labs	Cat# 017-000-121
Bovine Serum Albumin (IgG-Free, Protease-Free)	Jackson ImmunoResearch Labs	Cat# 001-000-161
ProLong® Gold Antifade Reagent with DAPI	Cell Signaling Technology	Cat# 8961S
Phalloidin-iFluor 594	Abcam	Cat# ab176757
Vector® TrueVIEW™ Autofluorescence Quenching Kit	Vector Laboratories	Cat# SP-8400-15
Sodium Citrate	Sigma-Aldrich	Cat# C8532
Tris	VWR Life Science	Cat# 0497
Signal Stain Ab Diluent	Cell Signaling Technology	Cat# 8112L
Animal-Free Blocking Solution (5X)	Cell Signaling Technology	Cat# 15019L
VECTASTAIN Elite ABC-HRP Kit (Peroxidase, Standard)	Vector Laboratories	Cat# PK-6100
ImmPACT DAB substrate kit	Vector Laboratories	Cat# SK-4105
Methyl Green	Vector Laboratories	Cat# H-3402
Hematoxylin QS	Vector Laboratories	Cat# H-3404
Dynabeads Protein A	Invitrogen	Cat# 10002D
Proteinase K	ThermoFisher	Cat# EO0491
Protease Inhibitor Cocktail	Sigma-Aldrich	Cat# P8340
Critical Commercial Assays		

Multi Tissue Dissociation Kit 2	Miltenyi Biotec	Cat# 130-110-203
Dead Cell Removal Kit	Miltenyi Biotec	Cat# 130-090-101
Red Blood Cell Lysis Solution (10X)	Miltenyi Biotec	Cat# 130-094-183
Anti-PE Microbeads	Miltenyi Biotec	Cat# 130-048-801
Nextera DNA Library Preparation Kit	Illumina	Cat# FC-121-1030
NEBNext High-Fidelity 2xPCR Master Mix	New England Biolabs	Cat# M0514S
Lipofectamine RNAiMAX Transfection Reagent	Life Technologies	Cat# 13778150
FuGENE HD Transfection Reagent	Promega	Cat# E2311
SsoAdvanced Universal SYBR Green Supermix	Bio-Rad	Cat# 172-5270
PowerUp SYBR Green Master Mix	Applied Biosystems	Cat# 100029283
MinElute Reaction Cleanup Kit	Qiagen	Cat# 28204
RNeasy Mini Kit	Qiagen	Cat# 74104
RNase-free DNase Set	Qiagen	Cat# 79254
Arcturus Picopure RNA Isolation Kit	Applied Biosystems	Cat# 12204-01
Microplate BCA Protein Assay Kit – Reducing Agent Compatible	Thermo Scientific	Cat# 23252
PureLink HiPure Plasmid Maxiprep Kit	Invitrogen	Cat# K210006
qPCR Lentivirus Titration Kit	abm	Cat# LV900
SimpleChIP Enzymatic Chromatin IP Kit (Magnetic Beads)	Cell Signaling	Cat# 9003
ChIP DNA Clean & Concentrator Kit	Zymo Research	Cat# D5201
Mycoplasma PCR Detection Kit	Applied Biological Materials	Cat# G238
Deposited Data		
In vivo mouse EPCAM-sorted endometrial epithelium RNA-seq dataset	Deposited to Gene Expression Omnibus	GEO accession number GSE129784
In vivo mouse EPCAM-sorted endometrial epithelium ATAC-seq dataset	Deposited to Gene Expression Omnibus	GEO accession number GSE129783
12Z cell line ATAC-seq dataset	Deposited to Gene Expression Omnibus	GEO accession number GSE129780
12Z cell line siRNA-transfection RNA-seq dataset	Deposited to Gene Expression Omnibus	GEO accession number GSE129782
12Z cell line siRNA and plasmid-transfection RNA-seq dataset	Deposited to Gene Expression Omnibus	GEO accession number GSE129779
12Z cell line ARID1A ChIP-seq dataset	Deposited to Gene Expression Omnibus	GEO accession number GSE129781
Experimental Models: Cell Lines		
Human: 12Z human endometriosis	Laboratory of Asgi Fazleabas	RRID: CVCL_0Q73
Human: Lenti-X^{TM} 293T embryonic kidney cells	Clontech	Cat# 632180; RRID: CVCL_0063
Experimental Models: Organisms/Strains		
Mouse: *Tg(Ltf-iCre)14Mmul*	Jackson Laboratory	Cat# 026030
Mouse: *(Gt)Rosa26LacZ*	Jackson Laboratory	Cat# 002955
Mouse: *(Gt)Rosa26Pik3ca*^{*H1047R}	Jackson Laboratory	Cat# 016977
Mouse: *Arid1a*^{fl}	Chandler et. al. 2015	N/A
Mouse: *Arid1a*^{V1068G}	Chandler et. al. 2013	N/A
Oligonucleotides		
ON-TARGETplus Non-targeting Pool	Dharmacon	Cat# D-001810
SMARTpool: ON-TARGETplus ARID1A siRNA	Dharmacon	Cat# L-017263-00

Primers for genotyping, see Table S1	This paper	N/A
Primers for qPCR, see Table S2	This paper	N/A
Recombinant DNA		
pBabe puro	AddGene	Cat# 1764
pBabe puro HA PIK3CA H1047R	AddGene	Cat# 12524
MISSION® pLKO.1-puro Non-Target shRNA Control Plasmid DNA	Sigma	Cat# SHC016
ARID1A MISSION® shRNA Plasmid DNA	Sigma	Cat# TRCN0000059091; NM_006015.3-7163s1c1
ARID1A MISSION® shRNA Plasmid DNA	Sigma	Cat# TRCN0000059090; NM_006015.3-1702s1c1
ARID1A MISSION® shRNA Plasmid DNA	Sigma	Cat# TRCN0000059089; NM_006015.3-2287s1c1
MISSION® Lentiviral Packaging Mix	Sigma	Cat# SHP001
Software and Algorithms		
Prism 7	Graphpad	www.graphpad.com
ImageJ 1.52k	National Institutes of Health	http://imagej.nih.gov/ij
FlowJo 10.4.2	TreeStar	www.flowjo.com
NIS Elements Advanced Research 4.30.02	Nikon	https://www.nikoninstruments.com
Adobe Illustrator CC 22.1	Adobe	www.adobe.com
Excel 16.16.2	Microsoft	https://office.microsoft.com/excel
R 3.4.3/3.5.1	R Core Team, 2018	https://www.r-project.org/
Trim Galore! 0.4.1	Krueger, F.	http://www.bioinformatics.babraham.ac.uk/projects/trim_galore/
FastQC 0.11.3	Andrews, 2010	http://www.bioinformatics.babraham.ac.uk/projects/fastqc
cutadapt 1.15	Martin, 2011	https://cutadapt.readthedocs.io/en/stable/
STAR 020201	Dobin et al,.2013	https://github.com/alexdobin/STAR
DESeq2 1.20.0	Love et al., 2014	https://bioconductor.org/packages/release/bioc/html/DESeq2.html
Bowtie2 2.2.6	Langmead and Salzberg, 2012	http://bowtie-bio.sourceforge.net/bowtie2/index.shtml
SAMtools 1.7	Li et al., 2009	http://www.htslib.org/doc/samtools.html
BEDtools 2.24.0	Quinlan and Hall, 2010	https://bedtools.readthedocs.io/en/latest/

MACS 2.1.0	Zhang et al., 2008	https://github.com/taoliu/MACS
csaw 1.12.0	Lun and Smyth, 2016	https://bioconductor.org/packages/release/bioc/html/csaw.html
ggplot2 3.0.0	Wickham, 2009	https://ggplot2.tidyverse.org/
preseqR 4.0.0	Deng, C. et al., 2018	https://cran.r-project.org/web/packages/preseqR/index.html
ATACseqQC 1.4.2	Ou et al., 2018	https://bioconductor.org/packages/release/bioc/html/ATACseqQC.html
Other		
MS Columns	Miltenyi Biotec	Cat# 130-042-201
Corning® Transwell® polycarbonate membrane cell culture inserts	Sigma-Aldrich	Cat# 3422
Cultrex PathClear Basement Membrane Extract	R & D Systems	Cat# 3432-005-01
Culture-Insert 4 Well in μ-Dish 35 mm high ibiTreat	ibidi	Cat# 80466
