# Supplementary material for: ARID1A and PI3-kinase pathway mutations in the endometrium drive epithelial transdifferentiation and collective invasion
Source: Nat Commun. 2019 Aug 7;10:3554. doi: 10.1038/s41467-019-11403-6 (PMC6686004; doi:10.1038/s41467-019-11403-6)
Supplement: Supplementary file 2 — Reporting Summary [file 41467_2019_11403_MOESM2_ESM.pdf]

nature research

Corresponding author(s): Ronald Chandler

Last updated by author(s): 6/12/2019

Reporting Summary

Nature Research wishes to improve the reproducibility of the work that we publish. This form provides structure for consistency and transparency in reporting. For further information on Nature Research policies, see Authors & Referees and the Editorial Policy Checklist.

Statistics

For all statistical analyses, confirm that the following items are present in the figure legend, table legend, main text, or Methods section.

n/a	Confirmed	
☐	☒	The exact sample size (*n*) for each experimental group/condition, given as a discrete number and unit of measurement
☐	☒	A statement on whether measurements were taken from distinct samples or whether the same sample was measured repeatedly
☐	☒	The statistical test(s) used AND whether they are one- or two-sided<br>*Only common tests should be described solely by name; describe more complex techniques in the Methods section.*
☐	☒	A description of all covariates tested
☐	☒	A description of any assumptions or corrections, such as tests of normality and adjustment for multiple comparisons
☐	☒	A full description of the statistical parameters including central tendency (e.g. means) or other basic estimates (e.g. regression coefficient) AND variation (e.g. standard deviation) or associated estimates of uncertainty (e.g. confidence intervals)
☐	☒	For null hypothesis testing, the test statistic (e.g. *F*, *t*, *r*) with confidence intervals, effect sizes, degrees of freedom and *P* value noted<br>*Give P values as exact values whenever suitable.*
☒	☐	For Bayesian analysis, information on the choice of priors and Markov chain Monte Carlo settings
☒	☐	For hierarchical and complex designs, identification of the appropriate level for tests and full reporting of outcomes
☒	☐	Estimates of effect sizes (e.g. Cohen's *d*, Pearson's *r*), indicating how they were calculated

Our web collection on statistics for biologists contains articles on many of the points above.

Software and code

Policy information about availability of computer code

Data collection	NIS Elements Advanced Research 4.30.02, Image Lab Software 5.1 build 8, QuantStudio Real-Time PCR Software v1.3, BD Accuri C6 Software version 1.0.264.21
Data analysis	Prism 7, FlowJo 10.4.2, Excel 16.16.2, R 3.4.3/3.5.1, Trim Galore! 0.4.1, FastQC 0.11.3, cutadapt 1.15, STAR 020201, DESeq2 1.20.0, Bowtie2 2.2.6, SAMtools 1.7, BEDtools 2.24.0, MACS 2.1.0, csaw 1.12.0, ggplot2 3.0.0, preseqR 4.0.0, ATACseqQC 1.4.2

For manuscripts utilizing custom algorithms or software that are central to the research but not yet described in published literature, software must be made available to editors/reviewers. We strongly encourage code deposition in a community repository (e.g. GitHub). See the Nature Research guidelines for submitting code & software for further information.

Data

Policy information about availability of data

All manuscripts must include a data availability statement. This statement should provide the following information, where applicable:

- Accession codes, unique identifiers, or web links for publicly available datasets
- A list of figures that have associated raw data
- A description of any restrictions on data availability

RNA-seq, ATAC-seq and ChIP-seq data that support the findings of this study have been deposited in GEO with the accession code GSE121198 (https://www.ncbi.nlm.nih.gov/geo/query/acc.cgi?acc=GSE121198)

Field-specific reporting

Please select the one below that is the best fit for your research. If you are not sure, read the appropriate sections before making your selection.

☒ Life sciences ☐ Behavioural & social sciences ☐ Ecological, evolutionary & environmental sciences

For a reference copy of the document with all sections, see nature.com/documents/nr-reporting-summary-flat.pdf

Life sciences study design

All studies must disclose on these points even when the disclosure is negative.

Sample size	For mouse studies, sample sizes within each genotype were chosen based on the proportions of animals with vaginal bleeding between each experimental group or a Kaplan-Meyer log rank test for survival differences. For cell line studies, all experiments were replicated at least twice.
Data exclusions	No data were excluded.
Replication	All experiments were reproduced, and all replication attempts were successful.
Randomization	Mice were allocated into groups based on PCR genotyping to confirm presence or absence of relevant alleles.
Blinding	Blinding was not relevant to this study, as samples were collected from mice on the basis of their genotype alone, and no additional procedures (surgery, drug treatment, etc) were performed.

Reporting for specific materials, systems and methods

We require information from authors about some types of materials, experimental systems and methods used in many studies. Here, indicate whether each material, system or method listed is relevant to your study. If you are not sure if a list item applies to your research, read the appropriate section before selecting a response.

Materials & experimental systems

n/a	Involved in the study	
☐	☒	Antibodies
☐	☒	Eukaryotic cell lines
☒	☐	Palaeontology
☐	☒	Animals and other organisms
☒	☐	Human research participants
☒	☐	Clinical data

Methods

n/a	Involved in the study	
☐	☒	ChIP-seq
☐	☒	Flow cytometry
☒	☐	MRI-based neuroimaging

Antibodies

Antibodies used

Rabbit monoclonal anti-ARID1A/BAF250A (D2A8U) Cell Signaling Technology Cat# 12354; RRID: AB_2637010; Lot# 2
Rabbit monoclonal anti-beta-Actin (D6A8) Cell Signaling Technology Cat# 8457; RRID:AB_10950489; Lot# 6
Rabbit monoclonal anti-Akt (pan) (C67E7) Cell Signaling Technology Cat# 4691; RRID: AB_915783; Lot# 20
Rabbit monoclonal anti-Phospho-Akt (Ser473) (D9E) Cell Signaling Technology Cat# 4060; RRID: AB_2315049; Lot# 19
Rabbit monoclonal anti-Phospho-S6 Ribosomal Protein (Ser235/236) (D57.2.2E) Cell Signaling Technology Cat# 4858; RRID: AB_2721245; Lot# 11
Rabbit monoclonal anti-BiP (C50B12) Cell Signaling Technology Cat# 3177; RRID: AB_10828008; Lot# 9
Rabbit monoclonal anti-E-Cadherin (24E10) Cell Signaling Technology Cat# 3195; RRID: AB_10694492; Lot# 13
Rabbit monoclonal anti-Vimentin (D21H3) Cell Signaling Technology Cat# 5741; RRID: AB_10695459; Lot# 5
Rabbit monoclonal anti-Grp94 (D6X2Q) Cell Signaling Technology Cat# 20292; RRID: AB_2722657; Lot# 1
Rabbit monoclonal anti-Snail (C15D3) Cell Signaling Technology Cat# 3879; RRID: AB_10828214; Lot# 12
Rabbit monoclonal anti-Slug (C19G7) Cell Signaling Technology Cat# 9585; RRID: AB_10828257; Lot# 6
Rabbit polyclonal anti-TWIST1 Cell Signaling Technology Cat# 46702; Lot# 1
Rabbit monoclonal anti-Estrogen Receptor Alpha (E115) Abcam Cat# ab32063; RRID: AB_732249; Lot# GR3184437-1
Rabbit monoclonal anti-Progesterone Receptor (SP2) Sigma-Aldrich Cat# SAB5500165; Lot# 160914LVD
Rabbit monoclonal anti-Cleaved Caspase-3 (Asp175) (D3E9) Cell Signaling Technology Cat# 9579; RRID: AB_10897512; Lot# 1
Rabbit polyclonal anti-Claudin-10 Thermo Fisher Scientific Cat# 38-8400; RRID: AB_2533386; Lot# QI215511
Rabbit polyclonal anti-ZO-1 Thermo Fisher Scientific Cat# 61-7300; RRID: AB_2533938; Lot# SL258826
Rat monoclonal anti-TROMA-I Developmental Studies Hybridoma Bank Cat# TROMA-I; RRID: AB_531826; Lot# 11/12/15
Rat monoclonal anti-mouse Ep-CAM Developmental Studies Hybridoma Bank Cat# G8.8; RRID: AB_2098655; Lot# 11/6/14
Mouse monoclonal anti-Actin alpha-Smooth Muscle Cy3 Sigma Cat# C6198; AB_476856; Lot# 042M4779
Goat anti-rabbit IgG, HRP-linked Antibody Cell Signaling Technology Cat# 7074; RRID: AB_2099233; Lot# 27
Donkey anti-Rabbit IgG, Biotin-SP-conjugated Jackson ImmunoResearch Labs Cat# 711-065-152; RRID: AB_2340593; Lot# 128874

Donkey anti-Rat IgG, Biotin-SP-conjugated Jackson ImmunoResearch Labs Cat# 712-065-153; RRID: AB_2315779; Lot# 125353
Anti-Rabbit IgG, Alexa Fluor 555-conjugated Antibody Thermo Fisher Scientific Cat# A-31572; RRID: AB_162543; Lot# 1806147
Anti-Rabbit IgG, Alexa Fluor 555-conjugated Antibody Thermo Fisher Scientific Cat# A-21428; RRID: AB_2535849; Lot#1774242
Anti-Rat IgG, Alexa Fluor 647-conjugated Antibody Thermo Fisher Scientific Cat# A-21247; RRID: AB_141778; Lot# 1810934
Anti-Rat IgG, Alexa Fluor 647-conjugated Antibody Jackson ImmunoResearch Labs Cat# 712-605-153; RRID: AB_2340694; Lot# 133374
Anti-CD326 (EpCAM)-PE, mouse (caa7-9G8) Miltenyi Biotec Cat# 130-102-265; Lot# 5170629682
Goat polyclonal anti-ICAM-1/CD54 R&D Systems Cat# AF796-SP; Lot# GRG0218121
Rabbit monoclonal anti-BRG1 (EPNCIR111A) Abcam Cat# ab110641; Lot# GR150844-17
Rabbit monoclonal anti-BRM (D9E8B) Cell Signaling Cat# 11966; Lot# 2
Mouse monoclonal anti-ARID1B (KMN1) Santa Cruz Biotechnology Cat# sc-32762; Lot# J0914
Mouse monoclonal anti-ARID1A (PSG3) Santa Cruz Biotechnology Cat# sc-32761; Lot# F0111
Rabbit monoclonal anti-ARID1B (E9J4T) Cell Signaling Cat# 92964; Lot# 1
Normal rabbit IgG Cat# 2729; Lot# 7

Validation

Rabbit monoclonal anti-ARID1A/BAF250A (D2A8U); validated by Cell Signaling Technology https://www.cellsignal.com/products/primary-antibodies/arid1a-baf250a-d2a8u-rabbit-mab/12354?_=1545322602265&Ntt=12354&tahead=true

Rabbit monoclonal anti-beta-Actin (D6A8); validated by Cell Signaling Technology https://www.cellsignal.com/products/primary-antibodies/b-actin-d6a8-rabbit-mab/8457?site-search-type=Products&N=4294956287&Ntt=d6a8&fromPage=plp

Rabbit monoclonal anti-Akt (pan) (C67E7); validated by Cell Signaling Technology https://www.cellsignal.com/products/primary-antibodies/akt-pan-c67e7-rabbit-mab/4691?site-search-type=Products&N=4294956287&Ntt=c67e7&fromPage=plp

Rabbit monoclonal anti-Phospho-Akt (Ser473) (D9E); validated by Cell Signaling Technology https://www.cellsignal.com/products/primary-antibodies/phospho-akt-ser473-d9e-xp-rabbit-mab/4060?site-search-type=Products&N=4294956287&Ntt=d9e&fromPage=plp

Rabbit monoclonal anti-Phospho-S6 Ribosomal Protein (Ser235/236) (D57.2.2E); validated by Cell Signaling Technology https://www.cellsignal.com/products/primary-antibodies/phospho-s6-ribosomal-protein-ser235-236-d57-2-2e-xp-rabbit-mab/4858?site-search-type=Products&N=4294956287&Ntt=d57.2.2e&fromPage=plp

Rabbit monoclonal anti-BiP (C50B12); validated by Cell Signaling Technology https://www.cellsignal.com/products/primary-antibodies/bip-c50b12-rabbit-mab/3177?site-search-type=Products&N=4294956287&Ntt=c50b12&fromPage=plp

Rabbit monoclonal anti-E-Cadherin (24E10); validated by Cell Signaling Technology https://www.cellsignal.com/products/primary-antibodies/e-cadherin-24e10-rabbit-mab/3195?site-search-type=Products&N=4294956287&Ntt=24e10&fromPage=plp

Rabbit monoclonal anti-Vimentin (D21H3); validated by Cell Signaling Technology https://www.cellsignal.com/products/primary-antibodies/vimentin-d21h3-xp-rabbit-mab/5741?_=1545328577353&Ntt=D21H3&tahead=true

Rabbit monoclonal anti-Grp94 (D6X2Q); validated by Cell Signaling Technology https://www.cellsignal.com/products/primary-antibodies/grp94-d6x2q-xp-rabbit-mab/20292?_=1545328588604&Ntt=D6X2Q&tahead=true

Rabbit monoclonal anti-Snail (C15D3); validated by Cell Signaling Technology https://www.cellsignal.com/products/primary-antibodies/snail-c15d3-rabbit-mab/3879?site-search-type=Products&N=4294956287&Ntt=c15d3&fromPage=plp

Rabbit monoclonal anti-Slug (C19G7); validated by Cell Signaling Technology https://www.cellsignal.com/products/primary-antibodies/slug-c19g7-rabbit-mab/9585?site-search-type=Products&N=4294956287&Ntt=c19g7&fromPage=plp

Rabbit polyclonal anti-TWIST1; validated by Cell Signaling Technology https://www.cellsignal.com/products/primary-antibodies/twist1-antibody/46702?_=1545328695200&Ntt=TWIST1&tahead=true

Rabbit monoclonal anti-Estrogen Receptor Alpha (E115); validated by AbCam https://www.abcam.com/estrogen-receptor-alpha-antibody-e115-chip-grade-ab32063.html

Rabbit monoclonal anti-Progesterone Receptor (SP2); validated by Sigma-Aldrich https://www.sigmaaldrich.com/catalog/product/sigma/sab5500165?lang=en&region=US

Rabbit monoclonal anti-Cleaved Caspase-3 (Asp175) (D3E9); validated by Cell Signaling Technology https://www.cellsignal.com/products/primary-antibodies/cleaved-caspase-3-asp175-d3e9-rabbit-mab/9579

Rabbit polyclonal anti-Claudin-10; validated Thermo Fisher Scientific https://www.thermofisher.com/antibody/product/Claudin-10-Antibody-Polyclonal/38-8400

Rabbit polyclonal anti-ZO-1; validated by Thermo Fisher Scientific https://www.thermofisher.com/antibody/product/ZO-1-Antibody-Polyclonal/61-7300

Rat monoclonal anti-TROMA-I; validated for IHC by Szeder, V., Grim, M., Halata, Z. & Sieber-Blum, M. Neural crest origin of mammalian Merkel cells. Dev Biol 253, 258-63 (2003).

Rat monoclonal anti-mouse Ep-CAM; validated by IHC by Borkowski, T.A., Nelson, A.J., Farr, A.G. & Udey, M.C. Expression of gp40, the murine homologue of human epithelial cell adhesion molecule (Ep-CAM), by murine dendritic cells. Eur J Immunol 26,

110-4 (1996).

Mouse monoclonal anti-Actin alpha-Smooth Muscle Cy3; validated by Sigma-Aldrich https://www.sigmaaldrich.com/catalog/product/sigma/c6198?lang=en&region=US

Goat anti-rabbit IgG, HRP-linked Antibody; validated by Cell Signaling Technology https://www.cellsignal.com/products/secondary-antibodies/anti-rabbit-igg-hrp-linked-antibody/7074?_=1545328712197&Ntt=7074&tahead=true

Donkey anti-Rabbit IgG, Biotin-SP-conjugated; validated by Jackson ImmunoResearch Labs https://www.jacksonimmuno.com/catalog/products/711-065-152

Donkey anti-Rat IgG, Biotin-SP-conjugated; validated by Jackson ImmunoResearch Labs https://www.jacksonimmuno.com/catalog/products/712-065-153

Anti-Rabbit IgG, Alexa Fluor 555-conjugated Antibody; validated by Thermo Fisher Scientific https://www.thermofisher.com/antibody/product/Donkey-anti-Rabbit-IgG-H-L-Highly-Cross-Adsorbed-Secondary-Antibody-Polyclonal/A-31572

Anti-Rabbit IgG, Alexa Fluor 555-conjugated Antibody; validated by Thermo Fisher Scientific https://www.thermofisher.com/antibody/product/Goat-anti-Rabbit-IgG-H-L-Cross-Adsorbed-Secondary-Antibody-Polyclonal/A-21428

Anti-Rat IgG, Alexa Fluor 647-conjugated Antibody; validated by Thermo Fisher Scientific https://www.thermofisher.com/antibody/product/Goat-anti-Rat-IgG-H-L-Cross-Adsorbed-Secondary-Antibody-Polyclonal/A-21247

Anti-Rat IgG, Alexa Fluor 647-conjugated Antibody; validated by Jackson ImmunoResearch Labs https://www.jacksonimmuno.com/catalog/products/712-605-153

Anti-CD326 (EpCAM)-PE, mouse (caa7-9G8); validated by Miltenyi Biotec https://www.miltenyibiotec.com/US-en/products/macs-flow-cytometry/antibodies/primary-antibodies/cd326-epcam-antibodies-mouse-caa7-9g8-1-10.html#pe-vio770:30-ug-in-1-ml

Goat polyclonal anti-ICAM-1/CD54; validated by R&D Systems https://www.rndsystems.com/products/mouse-icam-1-cd54-antibody_af796

Rabbit monoclonal anti-BRG1 (EPNCIR111A); validated by Abcam https://www.abcam.com/brg1-antibody-epncir111a-ab110641.html

Rabbit monoclonal anti-BRM (D9E8B); validated by Cell Signaling Technology https://www.cellsignal.com/products/primary-antibodies/brm-d9e8b-xp-rabbit-mab/11966

Mouse monoclonal anti-ARID1B (KMN1); validated by Santa Cruz Biotechnology https://www.scbt.com/scbt/product/arid1b-antibody-kmn1

Mouse monoclonal anti-ARID1A (PSG3); validated by Santa Cruz Biotechnology https://www.scbt.com/scbt/product/arid1a-antibody-psg3

Rabbit monoclonal anti-ARID1A (E9J4T); validated by Cell Signaling Technology https://www.cellsignal.com/products/primary-antibodies/arid1b-baf250b-e9j4t-rabbit-mab/92964?_=1554752160074&Ntt=ARID1B&tahead=true

Normal rabbit IgG; validated by Cell Signaling Technology https://www.cellsignal.com/products/primary-antibodies/normal-rabbit-igg/2729

Eukaryotic cell lines

Policy information about cell lines

Cell line source(s)	12Z endometrial epithelium cell line: Zeitvogel, A., Baumann, R. & Starzinski-Powitz, A. Identification of an invasive, N-cadherin-expressing epithelial cell type in endometriosis using a new cell culture model. Am J Pathol 159, 1839-52 (2001). Provided by the laboratory of Asgi Fazleabas.
Authentication	For the 12Z cell line validation study was performed by IDEXX BioResearch. The 12Z cell line has a unique profile not found in the current public databases.<br>The Lenti-X 293T cell line was purchased from Clontech.
Mycoplasma contamination	The 12Z and Lenti-X 293T cell lines tested negative for mycoplasma contamination. Testing was performed using the Mycoplasma PCR Detection Kit (Applied Biological Materials).
Commonly misidentified lines (See ICLAC register)	No commonly misidentified cell lines were used in this study.

Animals and other organisms

Policy information about studies involving animals; ARRIVE guidelines recommended for reporting animal research

Laboratory animals	Mouse, CD-1 strain, female, age 0-2 years.
Wild animals	The study did not involve wild animals.
Field-collected samples	The study did not involve samples collected from the field.
Ethics oversight	Michigan State University IACUC provided guidance on the protocol.

Note that full information on the approval of the study protocol must also be provided in the manuscript.

ChIP-seq

Data deposition

☒ Confirm that both raw and final processed data have been deposited in a public database such as GEO.

☒ Confirm that you have deposited or provided access to graph files (e.g. BED files) for the called peaks.

Data access links<br>*May remain private before publication.*	GEO accession: GSE121196 (https://www.ncbi.nlm.nih.gov/geo/query/acc.cgi?acc=GSE121196)<br>Reviewer access token: qhutwkqmfhqpdqj
Files in database submission	12Z_ChIP_Input_1st_R1.fastq.gz<br>12Z_ChIP_Input_2nd_R1.fastq.gz<br>12Z_ChIP_ARID1A_IP1_1st_R1.fastq.gz<br>12Z_ChIP_ARID1A_IP1_2nd_R1.fastq.gz<br>12Z_ChIP_ARID1A_IP2_1st_R1.fastq.gz<br>12Z_ChIP_ARID1A_IP2_2nd_R1.fastq.gz<br>12Z_ChIP_ARID1A_IP1_broad_peaks.filt.broadPeak<br>12Z_ChIP_ARID1A_IP2_broad_peaks.filt.broadPeak<br>12Z_ChIP_ARID1A_overlap_broad_peaks.filt.broadPeak
Genome browser session<br>(e.g. UCSC)	https://genome.ucsc.edu/s/reskejak/hg38_ChIPseq

Methodology

Replicates	Two ChIP replicates, each from 1x10^7 cells, were compared against input DNA. ChIP replicates called 61,393 and 61,307 broad peaks (FDR < 0.05), of which ~75.2% were retained as at least 50% overlapping by naive overlap, as defined by ENCODE.
Sequencing depth	All samples were sequenced on an Illumina NextSeq 500 in a single-end 75 bp read format in two technical replicates. ChIP replicates were sequenced to a total depth of 52.7M and 58.1M reads. Input DNA was sequenced to a total depth of 118.7M reads. PCR Duplication rates were 10.4% per library on average. Trimmed reads were uniquely mapped at rates of 73.50% and 72.06% for ChIP replicates and 73.69% for input DNA. Overall alignment of trimmed reads was >98.5%.
Antibodies	Rabbit monoclonal anti-ARID1A/BAF250A (D2A8U) Cell Signaling Technology Cat# 12354; RRID: AB_2637010; Lot# 2
Peak calling parameters	MACS2 was used to call broad peaks on individual IP replicates against input control with flags '--broad --broad-cutoff 0.05' to call broad peaks with FDR < 0.05 threshold. Replicates
Data quality	46,180 naive overlapping broad peaks were computed (FDR < 0.05), as defined by ENCODE. 12,627 naive overlapping broad peaks were above 5-fold enrichment.
Software	Base calling was done by Illumina NextSeq Control Software (NCS) v2.0 and output of NCS was demultiplexed and converted to FastQ format with Illumina Bcl2fastq v1.9.0. Technical replicate libraries were combined across flow cells and trimmed with cutadapt and Trim Galore! followed by quality control analysis via FastQC. Trimmed reads were aligned to GRCh38.p12 reference genome via Bowtie2 with flag '--very-sensitive'. Reads were then sorted and indexed with samtools. PCR duplicates were removed with Picard MarkDuplicates, and again sorted and indexed. MACS2 was used to call broad peaks with FDR < 0.05 threshold on each ChIP replicate against the input control, and the resulting peaks were repeat-masked by blacklist filtering. A naïve overlap peak set, as defined by ENCODE, was constructed by combining replicates and calling broad peaks on pooled BAM files followed by intersectBed to select for peaks of at least 50% overlap with each biological replicate.

Flow Cytometry

Plots

Confirm that:

☒ The axis labels state the marker and fluorochrome used (e.g. CD4-FITC).

☒ The axis scales are clearly visible. Include numbers along axes only for bottom left plot of group (a 'group' is an analysis of identical markers).

☒ All plots are contour plots with outliers or pseudocolor plots.

☒ A numerical value for number of cells or percentage (with statistics) is provided.

Methodology

Sample preparation	Mouse uteri were surgically removed and minced using scissors. Tissues were digested using the MACS Multi Tissue Dissociation Kit II (Miltenyi Biotec) for 80 min at 37º C. Digested tissues were strained through a 40 micrometer nylon mesh (ThermoFisher). The Red Cell Lysis Buffer (Miltenyi Biotec) was used to remove red blood cells. Dead cells removed using the MACS Dead Cell Removal Kit (Miltenyi Biotec), and EPCAM-positive cells were positively selected and purified using a PE-conjugated EPCAM antibody and anti-PE MicroBeads (Miltenyi Biotec), per the manufacturers' instructions.
Instrument	BD Accuri C6 flow cytometer
Software	BD Accuri C6 Software version 1.0.264.21 was used for data collection. FlowJo 10.4.2 software was used for data analysis.
Cell population abundance	EPCAM purified endometrial epithelium were on average 82% pure based on EPCAM-PE signal.
Gating strategy	Preliminary gating was defined by 0 SSC and 1.4x10^6 FSC. "Positive" EpCAM-PE staining was identified between 10^5 and 10^7 FL2-A.

☒ Tick this box to confirm that a figure exemplifying the gating strategy is provided in the Supplementary Information.
